# Effect of Periprocedural Myocardial Infarction After Initial Revascularization With Left Main PCI in Patients With Recent Myocardial Infarction

**DOI:** 10.1016/j.jscai.2022.100576

**Published:** 2023-04-03

**Authors:** Hao-Yu Wang, Bo Xu, Kefei Dou, Changdong Guan, Lei Song, Yunfei Huang, Rui Zhang, Lihua Xie, Weixian Yang, Yongjian Wu, Shubin Qiao, Yuejin Yang, Runlin Gao, Gregg W. Stone

**Affiliations:** a Department of Cardiology, Fuwai Hospital, National Center for Cardiovascular Diseases, Chinese Academy of Medical Sciences and Peking Union Medical College, Beijing, China; b Cardiometabolic Medicine Center, Fuwai Hospital, National Center for Cardiovascular Diseases, Chinese Academy of Medical Sciences and Peking Union Medical College, Beijing, China; c State Key Laboratory of Cardiovascular Disease, Beijing, China; d National Clinical Research Center for Cardiovascular Diseases, Beijing, China; e National Clinical Research Center for Cardiovascular Diseases, Fuwai Hospital, Chinese Academy of Medical Sciences, Shenzhen, Shenzhen, China; f The Zena and Michael A. Wiener Cardiovascular Institute, Icahn School of Medicine at Mount Sinai, New York, New York

**Keywords:** cardiac troponin, CK-MB, left main disease, periprocedural myocardial infarction, recent myocardial infarction

## Abstract

**Background:**

Periprocedural myocardial infarction (PMI) after percutaneous coronary intervention (PCI) for left main coronary artery disease (LMCAD) may be particularly deleterious in patients with recent myocardial infarction (MI). We sought to determine the rates and prognostic relevance of PMI using different definitions and biomarker thresholds after PCI for LMCAD in patients with recent MI.

**Methods:**

Between January 2004 and December 2016, 442 patients underwent PCI for LMCAD at a median of 3 days after presentation with MI. A total of 350 patients presented with elevated cardiac biomarker levels (349 with serial creatine kinase–myocardial band [CK-MB] and 219 with serial cardiac troponin I (cTnI) values) that were stable or falling before the PCI. In this cohort, PMI within 48 hours of PCI was adjudicated using Society for Cardiovascular Angiography & Interventions (SCAI), Academic Research Consortium 2 (ARC-2), and fourth Universal Definition of Myocardial Infarction (UDMI) criteria. The primary and secondary end points were 3-year rates of cardiovascular (CV) and all-cause death.

**Results:**

An incremental post-PCI rise in CK-MB starting at ≥10× the upper reference limit from baseline was significantly associated with 3-year CV death (adjusted hazard ratio [aHR], 7.96; 95% confidence interval [CI], 2.89-21.90), whereas CV death was not associated with any threshold elevation of cTnI. The frequencies of PMI according to the fourth UDMI, ARC-2, and SCAI definitions were 19.4%, 12.3%, and 8.6%, respectively. PMI by all 3 definitions was significantly associated with 3-year CV death, with the SCAI definition having the strongest relationship (aHR, 6.34; 95% CI, 2.47-16.27) compared with those of ARC-2 (aHR, 2.82; 95% CI, 1.15-6.96) and fourth UDMI (aHR, 2.65; 95% CI, 1.14-6.14).

**Conclusions:**

In patients with recent MI undergoing PCI for LMCAD, an incremental elevation in postprocedural CK-MB of ≥10× the upper reference limit as a stand-alone measure was strongly predictive of 3-year CV and all-cause death, whereas no cTnI elevations of any level were prognostic. All 3 contemporary PMI definitions in widespread use were associated with 3-year mortality after PCI in this high-risk cohort, with the SCAI definition having the strongest relationship with subsequent death.

## Highlights

• PMI after PCI for LMCAD may be particularly deleterious in patients with recent MI.
• An absolute incremental post-PCI increase in CK-MB ≥10× URL has impact on mortality.
• Supporting evidence of myocardial ischemia did not significantly change the relative utility of cardiac biomarkers.
• Supporting evidence of myocardial ischemia did not significantly change the relative utility of cardiac biomarkers.
• SCAI PMI definition was most strongly related to early and late mortality.

## Central Illustration

**Figure:**
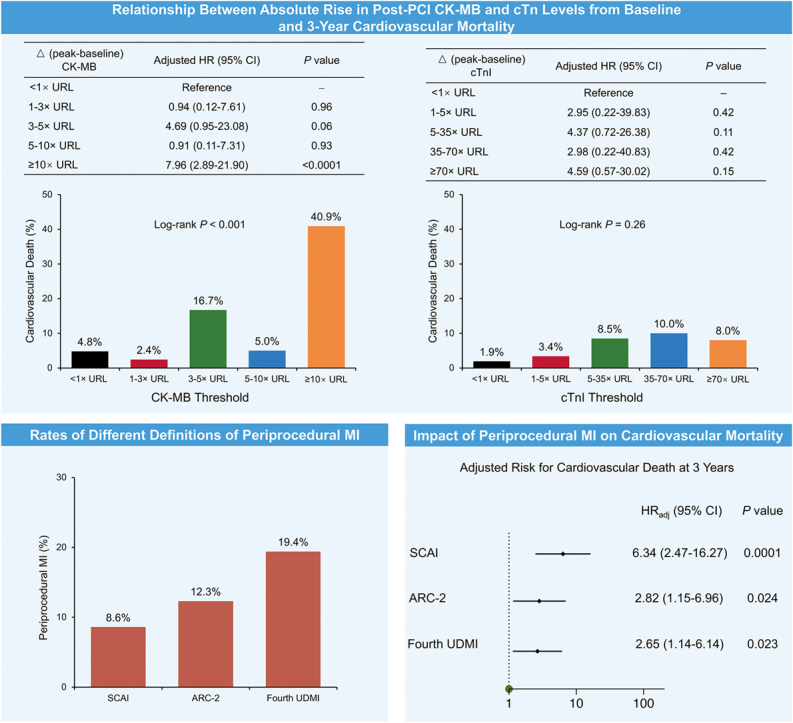

## Introduction

Current guidelines support percutaneous coronary intervention (PCI) with drug-eluting stents as an alternative to coronary artery bypass grafting (CABG) for treatment of stable patients with unprotected left main coronary artery disease (LMCAD) with low and intermediate anatomical complexity.^1^ Although these guidelines were based on randomized trials principally enrolling patients with stable ischemic heart disease, a substantial proportion of patients with LMCAD present with acute coronary syndromes at the index hospitalization.^2^^,^^3^ PCI is also frequently performed in patients with LMCAD and acute coronary syndrome, particularly in those with acute myocardial infarction (MI), especially if hemodynamic instability or cardiogenic shock is present.^4^^,^^5^ In such patients, periprocedural myocardial infarction (PMI) may be poorly tolerated given the large amount of myocardium subtended.

In this regard, controversy exists regarding the optimal definition of clinically relevant PMI.^6^^,^^7^ Uncertainties include the preferred cardiac biomarker (creatine kinase–myocardial band [CK-MB] or cardiac troponin [cTn]), the thresholds at which biomarker elevations become related with subsequent mortality, and whether inclusion of supporting evidence of myocardial ischemia (such as electrocardiographic [ECG] changes, procedural flow-limiting complications, regional wall motion abnormalities, or loss of viable myocardium) is necessary to support a prognostic relationship. Three definitions of PMI are in widespread use: the fourth Universal Definition of Myocardial Infarction (UDMI), Academic Research Consortium-2 (ARC-2), and Society for Cardiovascular Angiography & Interventions (SCAI) definitions. Each varies in their preferred biomarker, threshold for positivity, and requirements for supporting evidence of myocardial ischemia.^8^, ^9^, ^10^ Prior studies have noted substantial differences in the rates and prognostic effect of PMI after PCI for LMCAD according to the definition used.^11^, ^12^, ^13^ However, no prior study has examined the implications of each PMI definition (and individual biomarker thresholds) on mortality in patients with LMCAD and recent MI undergoing PCI, a particularly high-risk cohort in whom any additional myonecrosis may be especially deleterious.

Therefore, we sought to determine the following from a large prospective cohort of patients with recent MI undergoing PCI for LMCAD: (1) the post-PCI threshold of periprocedural myonecrosis using CK-MB and cTn that portends increased risk; (2) the frequency and clinical effect of PMI defined by the fourth UDMI, ARC-2, and SCAI definitions; and (3) the incremental utility of supporting evidence of myocardial ischemia on prognosis.

## Methods

### Study design and population

Consecutive patients who underwent unprotected PCI for LMCAD at Fu Wai Hospital (Chinese Academy of Medical Sciences and Peking Union Medical College, Beijing, China) have been prospectively enrolled in Fuwai LMCAD PCI registry since January 2004, with no exclusion criteria.^13^ This analysis was restricted to patients with recent MI (within 30 days) with baseline elevation of CK-MB and/or cTnI before PCI. The principal exclusion criterion for this analysis was the unavailability of pre-PCI and post-PCI serial cardiac biomarker measurements. In addition, the diagnosis of new PCI-related PMI cannot reliably be made in patients with only a single elevated biomarker or rising biomarkers before PCI that had not yet peaked. Thus, these patients were also excluded from this analysis (ie, serial pre-PCI measures with stable or falling biomarker levels were required). The registry was approved by the Fuwai Hospital Institutional Review Board and complied with the tenets of the Declaration of Helsinki. All patients provided written informed consent.

### Procedures, data collection, and patient follow-up

Percutaneous coronary intervention was performed according to standard practices (Appendix). Cardiac biomarkers (CK-MB and/or cTnI) were routinely assessed before PCI and at 8, 16, and 24 hours after PCI, and daily thereafter.^13^ Electrocardiography was performed before PCI, within 6 hours after PCI, and at discharge. Additional biomarker analysis, electrocardiography, and post-PCI echocardiography and/or angiography were performed if clinically indicated. Patients routinely received follow-up calls or outpatient visits at 1 month, 6 months, and 1 year and then annually through 3 years as described in the Appendix.

Data were prospectively entered into an electronic case report form by a trained research coordinator (C.D.G.) after review of medical records. Angiographic analyses, including Synergy Between Percutaneous Coronary Intervention With Taxus and Cardiac Surgery (SYNTAX) score, coronary lesions, and procedural flow-limiting complications, were performed at an independent core laboratory blinded to patient outcomes. The absolute incremental increase in post-PCI CK-MB or cTnI (Δ [peak − baseline]) were calculated as the difference between peak CK-MB or cTnI within 48 hours after PCI and the most recent pre-PCI value.

### Definitions of periprocedural myocardial injury and PMI

Two experienced cardiologists (W.X.Y., W.Y.W.) and 2 electrocardiographers (L.S., Y.F.H.) blinded to cardiac biomarker values and clinical outcomes independently adjudicated supporting evidence of new myocardial ischemia from ECG changes, imaging evidence (new loss of viable myocardium or new regional wall motion abnormality), and angiographic findings of procedural flow-limiting complications (including occlusion of a major epicardial artery or a side branch occlusion/thrombus, disruption of collateral flow, coronary dissection, slow flow or no-reflow, or distal embolization). Then, a separate independent clinical events committee adjudicated the extent of myocardial injury and PMI events according to the fourth UDMI, ARC-2, and SCAI definitions (Table 1 and Supplemental Table S1) after review of original source documents. CK-MB was used as the preferred biomarker for determination of PMI according to the SCAI definition (cTn was used if CK-MB was not available), whereas cTn was used as the preferred biomarker for determination of PMI according to the ARC-2 and fourth UDMI definitions (CK-MB was used if cTn was not available). Periprocedural myocardial injury as defined by fourth UDMI criteria and significant periprocedural myocardial injury as defined by ARC-2 criteria are depicted in detail in Supplemental Table S1. As a sensitivity analysis, PMI was also adjudicated according to the primary and secondary PMI definitions from the International Study of Comparative Health Effectiveness with Medical and Invasive Approaches (ISCHEMIA) (Supplemental Table S2).^14^

**Table 1:** Criteria for commonly used definitions for periprocedural myocardial infarction in patients with recent myocardial infarction.

Definition^a^	Time after procedure	Peak biomarker threshold	Supporting evidence required
SCAI	Within 48 h	In patients with elevated pre-PCI biomarker levels that are stable or falling: i) An absolute rise in CK-MB from baseline of ≥10× URL (used preferentially if available) or absolute increase in cTn from baseline of ≥70× URL), or ii) An absolute rise in CK-MB of ≥5× URL (used preferentially if available) or absolute increase in cTn from baseline of ≥35× URL	i) None ii) ECG: New pathological Q waves in ≥2 contiguous leads or new persistent LBBB
ARC-2	Within 48 h	In patients with elevated pre-PCI biomarker levels that are stable or falling: An absolute rise in cTn from baseline of ≥35× URL (used preferentially if available) or an absolute rise in CK-MB from baseline of ≥5× URL	One or more of the following: ECG: New significant Q waves or equivalent Angiographic: Occlusion of a major epicardial artery or a side branch, major coronary dissection, disruption of collateral flow, distal embolization, or persistent slow flow or no reflow Imaging: New substantial loss of myocardium on imaging
Fourth UDMI	Within 48 h	In patients with elevated pre-PCI biomarker levels that are stable or falling: cTn rise by >20% from baseline and the absolute cTn value must still be >5× URL (used preferentially if available), or CK-MB rise by >20% from baseline and the absolute CK-MB value must still be >5× URL	One or more of the following: ECG: New ischemic ECG changes (ST segments) or development of new pathological Q waves Angiographic: Occlusion of a major epicardial artery or a side branch, major coronary dissection, disruption of collateral flow, distal embolization, or persistent slow flow or no reflow Imaging: Evidence of new loss of viable myocardium or new regional wall motion abnormality in a pattern consistent with an ischemic etiology

ARC-2, Academic Research Consortium-2; CK-MB, creatine kinase–myocardial band; cTn, cardiac troponin; ECG, electrocardiographic; LBBB, left bundle branch block; PCI, percutaneous coronary intervention; SCAI, Society for Cardiovascular Angiography & Interventions; UDMI, Universal Definition of Myocardial Infarction; URL, upper reference limit.

a For the Society for Cardiovascular Angiography & Interventions definition, creatine kinase–myocardial band is used preferentially if available, cardiac troponin otherwise. For the Academic Research Consortium-2 and fourth Universal Definition of Myocardial Infarction definitions, cardiac troponin is used preferentially if available, creatine kinase–myocardial band otherwise. In the Fuwai angiographic core laboratory, a major side branch occlusion was defined as a branch supplying the left ventricle (including diagonal, posterolateral, posterior descending, obtuse marginal, and septal branches) that was ≥1.5 mm in diameter; major coronary dissection was defined as dissection in the target vessel greater than type B from National Heart, Lung, and Blood Institute classification; disruption of collateral flow was defined as reduction in collateral flow by ≥1 grades (Rentrop classification); distal embolization was defined as the appearance of an abrupt cutoff in the distal vessel (or in a side branch ≥1.5 mm) after percutaneous coronary intervention; persistent slow flow or no reflow was defined as markedly delayed flow (thrombolysis in myocardial infarction grade 2 for slow flow, thrombolysis in myocardial infarction grade 0 or 1 for no reflow) in a target vessel with minimal (<30%) residual stenosis at the stented/scaffolded segment and no evidence of flow-limiting dissection.

### Clinical end points

The primary end point was the covariate-adjusted 3-year rate of cardiovascular (CV) mortality. The key secondary end point was the covariate-adjusted 3-year rate of all-cause death. CV death was classified by the clinical events committee according to ARC-2 criteria as death that was due to a clear CV cause, was procedure-related, or was due to an undetermined cause. End point definitions are reported in the Appendix.

### Statistical analysis

Continuous variables were compared using the *t* test. Categorical variables were compared using the χ^2^ or Fisher exact test as appropriate. Follow-up event rates were estimated by the Kaplan-Meier method and were compared between groups using Cox proportional regression. Unadjusted and adjusted hazard ratios (aHRs) for the associations between prespecified commonly used intervals of postprocedural CK-MB elevations normalized to the upper reference limit (URL) (<1×, ≥1× to <3×, ≥3× to <5×, ≥5× to <10×, and ≥10×) and cTnI elevations normalized to the URL (<1×, ≥1× to <5×, ≥5× to <35×, ≥35× to <70×, and ≥70×) and 3-year CV and all-cause mortality were determined. The prognostic significance of PMI according to different definitions (SCAI, ARC-2, and fourth UDMI) was similarly assessed. The covariates in the multivariable Cox regression models were selected according to their historical associations with periprocedural myonecrosis and death in patients with recent MI undergoing PCI and included age, sex, smoking, hypertension, diabetes, prior MI, left ventricular dysfunction with ejection fraction of <40%, and multivessel disease. In addition, we examined the risk of mortality according to biomarker thresholds of CK-MB (≥5× URL or ≥10× URL) or cTnI (≥5× URL or ≥35× URL) with or without supporting evidence of new myocardial ischemia, representing common cutoffs from the fourth UDMI, ARC-2, and SCAI definitions of PMI. Additional exploratory analyses were performed to evaluate the effect of PMI in relation to important subgroups (timing of recent MI of >24 vs ≤24 hours and presence of pulmonary edema or cardiogenic shock).

## Results

### Study population

**Figure 1:**
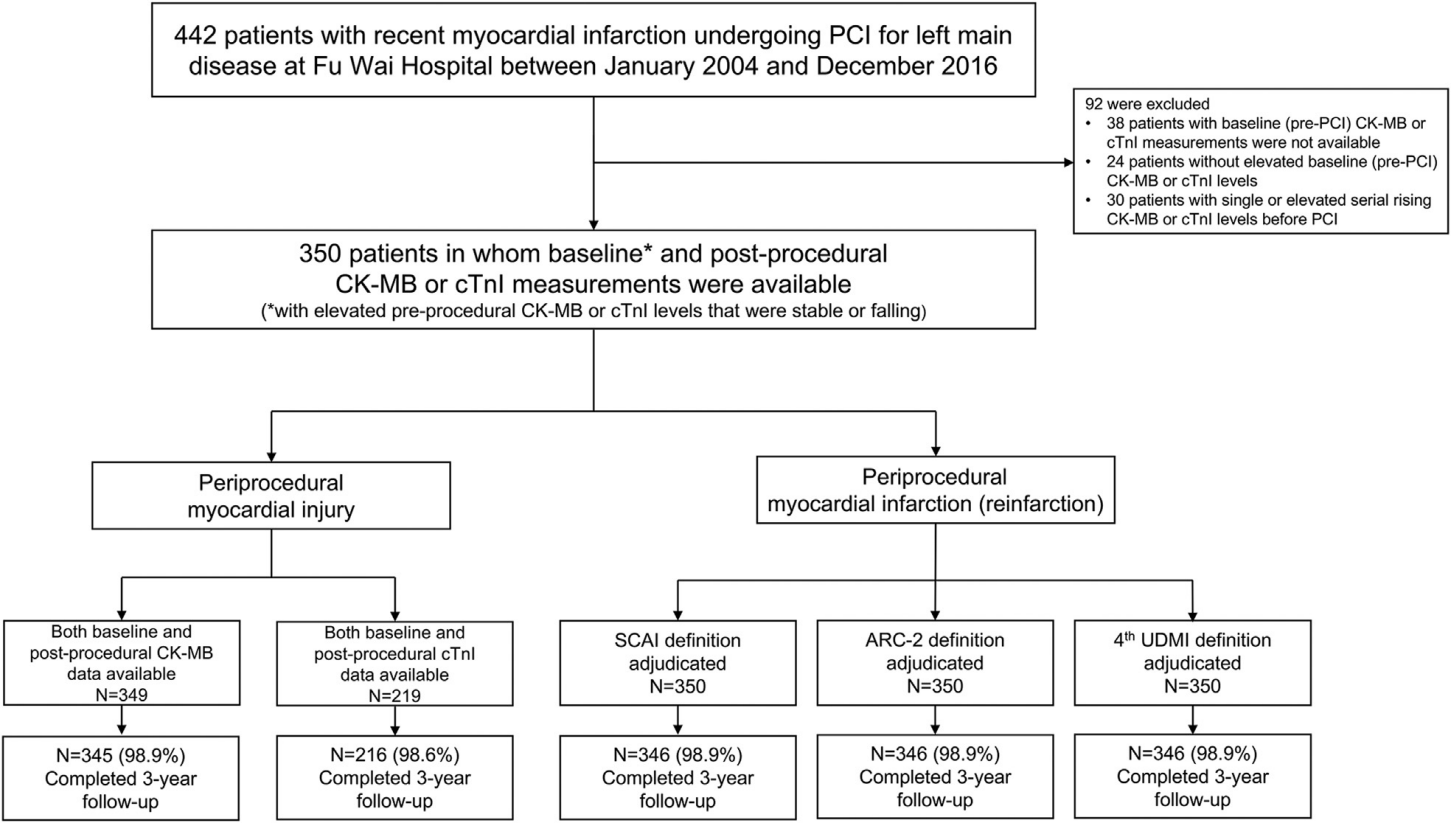
**Study flowchart.** The study population comprised 350 patients (349 with serial creatine kinase–myocardial band [CK-MB] and 219 with serial cardiac troponin I [cTnI] measures) with recent myocardial infarction undergoing percutaneous coronary intervention (PCI) for left main coronary artery disease in whom baseline CK-MB or cTnI were elevated and stable or falling before the PCI. ARC-2, Academic Research Consortium 2; SCAI, Society for Cardiovascular Angiography & Interventions; UDMI, Universal Definition of Myocardial Infarction.

Between January 2004 and December 2016, 4625 patients with LMCAD who underwent PCI were enrolled into the registry, 442 of whom experienced recent MI (within 30 days). Ninety-two patients were excluded for lack of preprocedural (n = 38) or elevated (n = 24) biomarker measurements or only a single or serial rising CK-MB or cTnI levels that had not peaked before PCI (n = 30). The remaining 350 patients in whom baseline serial CK-MB or cTnI values were elevated and were stable or falling constituted this study population (Figure 1), in which the median number of cardiac biomarker samples obtained before PCI for LMCAD was 3 (interquartile range [IQR], 2-4). Of these, 349 (99.7%) and 219 (62.6%) patients had serial CK-MB and cTnI measurements, respectively.

### Baseline and procedural characteristics

As shown in Tables 2 and 3, the mean age was 61.7 ± 12.1 years; 76.9% (269/350) of patients were men, and 23.1% (81/350) reported diabetes. The qualifying recent MI type was non–ST-segment elevation MI in 64.0% (224/350) and ST-segment elevation MI in 36.0%, and 9.7% (34/350) of patients presented with pulmonary edema or cardiogenic shock. The median (IQR) duration from hospital presentation with MI to PCI was 3 (1-8) days; 35.4% of patients underwent PCI within 24 hours of presentation. The mean SYNTAX score (core laboratory analysis) was 24.3 ± 7.6. Radial access and intravascular ultrasound were used in 78.0% and 38.6% of the PCI procedures, respectively.

**Table 2:** Baseline clinical and angiographic characteristics.

Characteristics	Values
Clinical	n = 350
Age, y	61.7 ± 12.1
Male sex	76.9 (269/350)
Body mass index, kg/m^2^	25.6 ± 3.7
Diabetes mellitus	23.1 (81/350)
Hypertension	53.4 (187/350)
Hyperlipidemia	57.4 (201/350)
Renal insufficiency^a^	18.6 (65/350)
Current smoking	35.7 (125/350)
Family history of coronary artery disease	17.7 (62/350)
Peripheral vascular disease	6.0 (21/350)
Prior PCI	26.3 (92/350)
Prior MI^b^	12.9 (45/350)
Prior stroke	15.7 (55/350)
Creatinine clearance, mL/min	84.9 ± 31.6
Systolic blood pressure on admission, mm Hg	124.4 ± 19.3
Diastolic blood pressure on admission, mm Hg	74.1 ± 11.7
Heart rate on admission (per min)	72.7 ± 14.2
Left ventricular ejection fraction, %	60 ± 34
Left ventricular ejection fraction <50%	19.0 (66/348)
Killip class	
I	79.7 (279/350)
II	10.6 (37/350)
III (pulmonary edema)	4.0 (14/350)
IV (cardiogenic shock)	5.7 (20/350)
Clinical presentation	
Non–ST-segment elevation MI	64.0 (224/350)
ST-segment elevation MI	36.0 (126/350)
Time from recent MI admission to PCI, d	3 (1-8)
≤24 h	35.4 (124/350)
≤72 h	52.3 (183/350)
Angiographic (core laboratory)	n = 350
No. of diseased non–left main coronary arteries^c^	
0	16.3 (57/350)
1	33.4 (117/350)
2	31.7 (111/350)
3	18.6 (65/350)
Left main lesion type	
De novo	96.6 (338/350)
Restenosis	3.4 (12/350)
Left main lesion location	
Ostium	11.7 (41/350)
Shaft	5.4 (19/350)
Distal bifurcation	82.9 (290/350)
Complex left main distal bifurcation lesion	27.2 (79/350)
Medina 1,1,1 bifurcation	78.5 (62/79)
Medina 0,1,1 bifurcation	21.5 (17/79)
Left main moderate or severe calcification	13.1 (46/350)
Left main thrombus-containing lesions	7.4 (26/350)
Left main total occlusions^d^	12.3 (43/350)
Left main lesion length, mm	28.7 ± 19.3
SYNTAX score	24.3 ± 7.6
Low (≤22)	42.3 (148/350)
Intermediate (23-32)	46.6 (163/350)
High (≥33)	11.1 (39/350)

Values are mean ± SD or % (n/N).

MI, myocardial infarction; PCI, percutaneous coronary intervention; SYNTAX, Synergy Between Percutaneous Coronary Intervention With Taxus and Cardiac Surgery.

a Baseline creatinine clearance calculated using the Cockcroft-Gault equation <60 mL/min.

b Previous myocardial infarction means before this presentation with myocardial infarction.

c Diameter stenosis of ≥50% by visual estimation by angiographic core laboratory assessment.

d Defined as percent diameter stenosis of ≥99% and thrombolysis in myocardial infarction flow grade 0 or 1.

**Table 3:** Procedural characteristics, techniques, and outcomes.

Procedural characteristics	Values
Transradial intervention	78.0 (273/350)
Intravascular ultrasound guidance	38.6 (135/350)
Stent implantation	97.7 (342/350)
Total number of stents per patient	2.2 ± 1.2
Type of stents	
Bare metal	2.6 (9/342)
Drug-eluting	97.4 (333/342)
Total stent length per patient, mm	43.9 ± 27.1
Total number of stents in left main	1.7 ± 0.8
Total stent length in left main, mm	31.8 ± 19.0
Left main mean stent diameter, mm	3.46 ± 0.47
Left main bifurcation treated with a provisional crossover technique	77.9 (226/290)
Left main bifurcation treated with a planned 2-stent technique	22.1 (64/290)
Culotte	12.5 (8/64)
Crush	70.3 (45/64)
T-stent	10.9 (7/64)
V or simultaneous kissing stent	6.3 (4/64)
Left main bifurcation with final kissing balloon inflation	55.9 (162/290)
Postdilation performed	74.3 (260/350)
No. of non-left main lesions treated per patient	0.5 ± 0.7
Intra-aortic balloon pump utilization	21.7 (76/350)
Placed before PCI	55.3 (42/76)
Placed after PCI	44.7 (34/76)
Residual SYNTAX score	4.8 ± 5.9

Values are mean ± SD or % (n/N).

PCI, percutaneous coronary intervention; SYNTAX, Synergy Between Percutaneous Coronary Intervention With Taxus and Cardiac Surgery.

### Periprocedural myocardial injury and infarction

Distributions of the incremental threshold increases in CK-MB and cTnI from baseline are shown in Supplemental Table S3. Any new elevations (incremental threshold rise ≥1× URL) were noted in 27.5% of cases with CK-MB assessments and 51.1% of cases with cTnI assessments. The incidence of periprocedural myocardial injury was 44.3% as defined by the fourth UDMI and 9.4% as defined by ARC-2 criteria. The frequencies of PMI according to the fourth UDMI, ARC-2, and SCAI definitions were 19.4% (n = 68), 12.3% (n = 43) and 8.6% (n = 30), respectively (Central Illustration and Supplemental Table S4). Overlap between PMI according to the different definitions is shown in Supplemental Table S5.

**Central Illustration:**
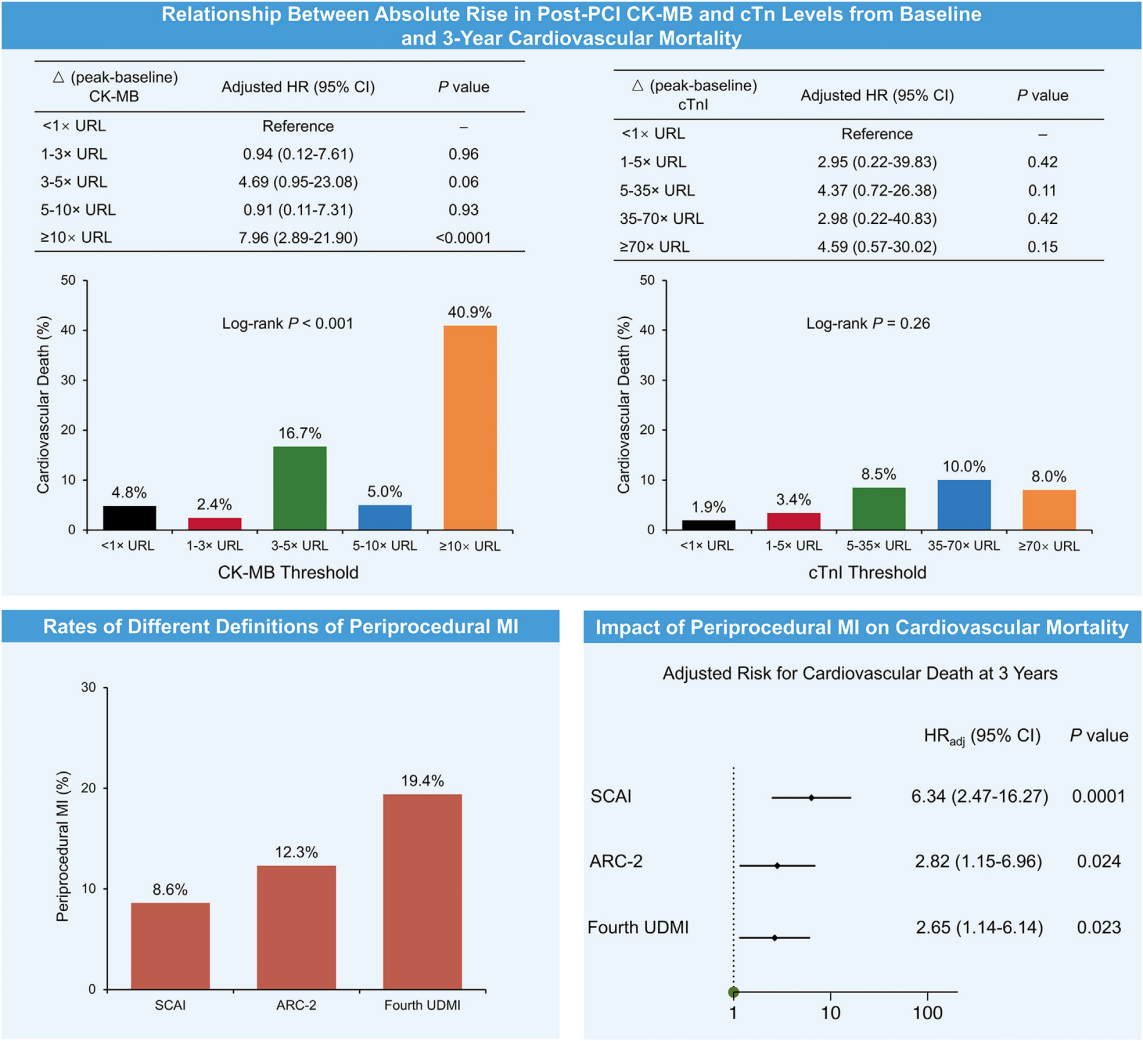
Prognostic effect of absolute rise in post–percutaneous coronary intervention (PCI) biomarker levels from baseline and different definitions of periprocedural myocardial infarction (MI) for 3-year cardiovascular death. (**Upper**) Relationship between the interval absolute rises of post-PCI creatine kinase–myocardial band (CK-MB) and cardiac troponin (cTn) I elevation from baseline and 3-year cardiovascular mortality. **(Lower left)** Frequency of periprocedural MI according to Society for Cardiovascular Angiography & Interventions (SCAI), Academic Research Consortium-2 (ARC-2), and fourth Universal Definition of Myocardial Infarction (UDMI) definitions. (**Lower right**) Effect of periprocedural MI defined by SCAI, ARC-2, and fourth UDMI definitions on 3-year cardiovascular death. HR, hazard ratio; HR_adj_, adjusted hazard ratio; URL, upper reference limit.

### Relationship between biomarker-defined periprocedural myonecrosis and subsequent mortality

**Figure 2:**
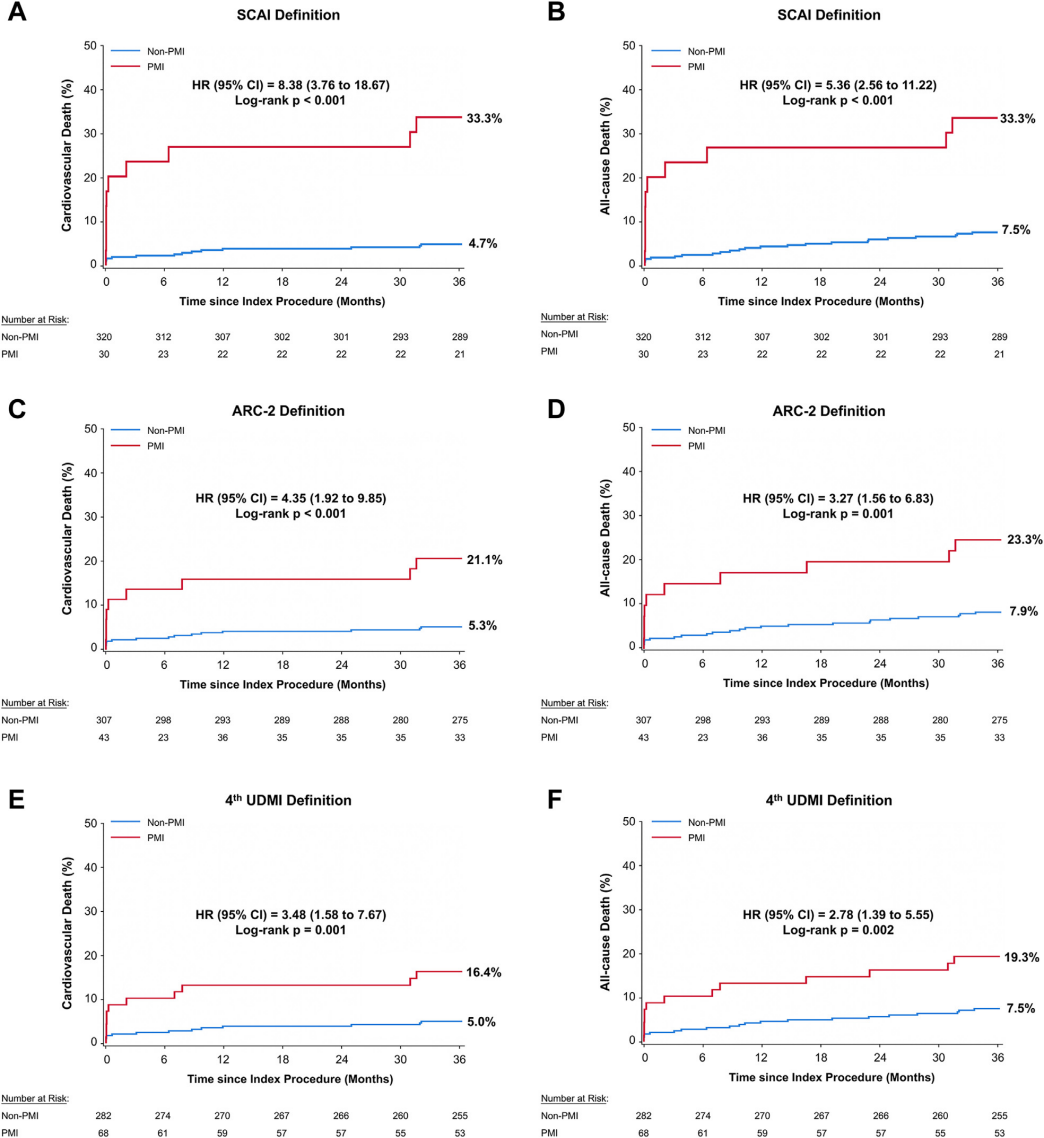
**Time-to-event curves for 3-year cardiovascular and all-cause death in patients with versus without periprocedural myocardial infarction (PMI) according to various contemporary definitions.** Kaplan-Meier curves for cardiovascular death (A, C, and E) and all-cause death (B, D, and F) in patients with versus without PMI according to Society for Cardiovascular Angiography & Interventions (SCAI), Academic Research Consortium-2 (ARC-2), and fourth Universal Definition of Myocardial Infarction (UDMI) definitions, respectively. HR, hazard ratio.

Three-year follow-up data were available in 346 (98.9%) of 350 patients. During the median 3.1-year follow-up (IQR, 3.1-3.1), there were 34 all-cause deaths (Kaplan-Meier estimated rate, 9.8%), including 25 CV deaths (7.2%). Other events are shown in Supplemental Table S4. Relationships between the absolute increase in postprocedural peak biomarker from baseline and mortality are shown in Table 4 and Figure 2. Only an absolute incremental rise after PCI in CK-MB of ≥10× URL (compared with <URL) was associated with an increased risk of 3-year CV death (40.9% vs 4.8%; aHR, 7.96; 95% CI, 2.89-21.90; *P* < .0001) and all-cause death (40.9% vs 8.0%; aHR, 5.43; 95% CI, 2.23-13.21; *P* = .0002), compared with an absolute incremental rise after PCI in CK-MB of <1× URL (Table 4 and Supplemental Figure S1). No significant relationship between any cTnI elevation after PCI was present with either 3-year CV or all-cause mortality. Similarly, neither periprocedural myocardial injury as defined by the fourth UDMI criteria nor ARC-2 criteria after PCI were related to 3-year CV death or all-cause death (Supplemental Table S6).

**Table 4:** Absolute increase in postprocedural peak biomarker from baseline and 3-year cardiovascular and all-cause mortality.

Biomarker threshold	% (n/N)	Unadjusted HR (95% CI)	*P* value	Adjusted HR^a^ (95% CI)	*P* value
Cardiovascular mortality					
Δ (peak − baseline) CK-MB					
<1× URL	4.8 (12/253)	Reference	–	Reference	–
1-3× URL	2.4 (1/42)	0.50 (0.06-3.81)	.50	0.94 (0.12-7.61)	.96
3-5× URL	16.7 (2/12)	3.91 (0.88-17.49)	.07	4.69 (0.95-23.08)	.06
5-10× URL	5.0 (1/20)	1.06 (0.14-8.09)	.96	0.91 (0.11-7.31)	.93
≥10× URL	40.9 (9/22)	10.68 (4.49-25.40)	<.0001	7.96 (2.89-21.90)	<.0001
*P* value for trend			<.0001		.001
Δ (peak − baseline) cTnI					
<1× URL	1.9 (2/107)	Reference	–	Reference	–
1-5× URL	3.4 (1/30)	1.84 (0.17-20.30)	.62	2.95 (0.22-39.83)	.42
5-35× URL	8.5 (4/47)	5.03 (0.92-27.49)	.06	4.37 (0.72-26.38)	.11
35-70× URL	10.0 (1/10)	5.72 (0.52-63.12)	.16	2.98 (0.22-40.83)	.42
≥70× URL	8.0 (2/25)	4.41 (0.62-31.32)	.14	4.59 (0.57-30.02)	.15
*P* value for trend			.36		.48
All-cause mortality					
Δ (peak − baseline) CK-MB					
<1× URL	8.0 (20/253)	Reference	–	Reference	–
1-3× URL	4.8 (2/42)	0.60 (0.14-2.55)	.58	1.04 (0.23-4.63)	.96
3-5× URL	16.7 (2/12)	2.36 (0.55-10.08)	.25	2.72 (0.61-12.05)	.19
5-10× URL	5.0 (1/20)	0.63 (0.09-4.70)	.65	0.59 (0.08-4.46)	.61
≥10× URL	40.9 (9/22)	6.59 (3.00-14.50)	<.0001	5.43 (2.23-13.21)	.0002
*P* value for trend			<.0001		.003
Δ (peak − baseline) cTnI					
<1× URL	3.8 (4/107)	Reference	–	Reference	–
1-5× URL	6.7 (2/30)	1.85 (0.34-10.12)	.48	2.36 (0.41-13.70)	.34
5-35× URL	17.3 (8/47)	5.13 (1.54-17.04)	.008	4.32 (1.27-14.77)	.019
35-70× URL	20.0 (2/10)	5.89 (1.08-32.18)	.041	3.65 (0.62-21.53)	.15
≥70× URL	8.0 (2/25)	2.20 (0.40-12.03)	.36	1.94 (0.34-11.20)	.46
*P* value for trend			.07		.12

Event rates are Kaplan-Meier estimated rates (%) at 3 years (number of events/denominator at baseline). Adjusted hazard ratios and 95% confidence intervals were generated using multivariable Cox regression analysis.

CK-MB, creatine kinase–myocardial band; cTnI, cardiac troponin I; HR, hazard ratio; URL, upper reference limit.

a Model adjusted for age, sex, current smoking, hypertension, diabetes mellitus, prior myocardial infarction, left ventricular dysfunction with ejection fraction of <40%, and 2- or 3-vessel disease.

### Effect of PMI according to various definitions on subsequent mortality risk

The 3-year risks of CV and all-cause death after PMI according to the 3 major definitions in widespread use are shown in Table 5 and Figure 2. PMI by all 3 definitions was independently associated with subsequent mortality. For both CV and all-cause death, the strongest relationship was with PMI according to the SCAI definition (CV death: aHR, 6.34; 95% CI, 2.47-16.27; *P* = .0001; all-cause death: aHR, 4.48; 95% CI, 1.95-10.27; *P* = .0004) and the weakest relationship was with the fourth UDMI (CV death: aHR, 2.65; 95% CI, 1.14-6.14; *P* = .023; all-cause death: aHR, 2.33; 95% CI, 1.13-4.80; *P* = .022) (Central Illustration). Furthermore, PMI defined by ARC-2 only (after excluding those also meeting SCAI PMI criteria) and PMI defined by the fourth UDMI only (after excluding those also meeting SCAI or ARC-2 PMI criteria) were not significantly associated with 3-year CV or all-cause mortality (Supplemental Table S7).

**Table 5:** Hazard ratios for 3-year cardiovascular and all-cause mortality in patients with versus without periprocedural myocardial infarction according to various contemporary definitions.

PMI definition	Patients with PMI	Patients without PMI	Unadjusted HR (95% CI)	*P* value	Adjusted HR^a^ (95% CI)	*P* value
Cardiovascular mortality						
SCAI definition	33.3 (10/30)	4.7 (15/320)	8.38 (3.76-18.67)	<.0001	6.34 (2.47-16.27)	.0001
ARC-2 definition	21.1 (9/43)	5.3 (16/307)	4.35 (1.92-9.85)	.0004	2.82 (1.15-6.96)	.024
Fourth UDMI definition	16.4 (11/68)	5.0 (14/282)	3.48 (1.58-7.67)	.002	2.65 (1.14-6.14)	.023
All-cause mortality						
SCAI definition	33.3 (10/30)	7.6 (24/320)	5.36 (2.56-11.22)	<.0001	4.48 (1.95-10.27)	.0004
ARC-2 definition	23.3 (10/43)	7.9 (24/307)	3.27 (1.56-6.83)	.002	2.46 (1.12-5.41)	.026
Fourth UDMI definition	19.3 (13/68)	7.5 (21/282)	2.78 (1.39-5.55)	.004	2.33 (1.13-4.80)	.022

Event rates are Kaplan-Meier estimated rates (%) at 3 years (number of events/denominator at baseline). Adjusted hazard ratios and 95% confidence intervals were generated using multivariable Cox regression analysis.

ARC-2, Academic Research Consortium-2; HR, hazard ratio; PMI, Periprocedural myocardial infarction; SCAI, Society for Cardiovascular Angiography & Interventions; UDMI, Universal Definition of Myocardial Infarction.

a Model adjusted for age, sex, current smoking, hypertension, diabetes mellitus, prior myocardial infarction, left ventricular dysfunction with ejection fraction <40%, and 2- or 3-vessel disease.

### Effect of supporting evidence of myocardial ischemia

The unadjusted hazard ratios for the relationship between cTnI elevations and subsequent morality were somewhat improved when supporting evidence of myocardial ischemia was present. However, the adjusted relationships between cTnI elevations and CV and all-cause mortality were still not significant even when myocardial ischemia was present (Supplemental Tables S8 and S9). Conversely, CK-MB elevations of ≥10× URL were independently associated with subsequent CV and all-cause mortality with or without accompanying evidence of myocardial ischemia (Supplemental Tables S10 and S11).

### Subgroup and sensitivity analyses

The associations between PMI per the 3 main definitions and 3-year CV and all-cause mortality were consistently observed in patients with recent MI of ≤24 and >24 hours and in those with and without pulmonary edema or cardiogenic shock (Supplemental Tables S12 and S13). PMI was less common with the ISCHEMIA primary definition compared with its secondary definition (11.1% vs 20.6%). Moreover, the ISCHEMIA primary PMI definition compared with the secondary PMI was more strongly related to 3-year CV and all-cause death (Supplemental Table S14).

## Discussion

This study is, to our knowledge, the first comprehensive evaluation of various biomarkers and PMI definitions in high-risk patients undergoing PCI for LMCAD at a median of 3 days after presentation with MI. As summarized in the Central Illustration, the principal findings are as follows:

1. As a stand-alone measure, only an absolute incremental rise in post-PCI CK-MB from baseline by ≥10× URL was an independent determinant of 3-year CV and all-cause mortality, whereas any threshold elevation of cTnI (even ≥70× URL) was not associated with outcomes.
2. Ancillary ECG and imaging evidence of myocardial ischemia did not significantly change the relative utility of CK-MB versus cTnI biomarkers.
3. With systematic biomarker assessments, PMI after PCI was relatively frequent in this study cohort, although the rates of PMI varied greatly depending on the specific definitions used. PMI was more than twice as frequent with the fourth UDMI (19.4%) compared with the SCAI definition (8.6%).
4. All definitions of PMI were independently associated with 3-year CV and all-cause mortality after PCI in patients with LMCAD presenting with recent MI; however, the SCAI definition showed the greatest hazard for reduced survival.
5. When patients with SCAI PMIs were removed from those with fourth UDMI and ARC-2 PMIs, the smaller infarctions left behind were no longer prognostic.

Compared with those with chronic coronary syndromes, patients with recent MI have higher risks of adverse CV events and mortality after revascularization.^15^ Presentation of LMCAD with acute MI is not uncommon, and these patients represent a high-risk cohort for revascularization owing to frequent left ventricular dysfunction and the large amount of myocardium jeopardized.^2^^,^^3^ Additional myocardial injury arising from the procedure itself in such patients may be especially deleterious.^12^^,^^13^ However, the diagnosis of myocardial injury or PMI after PCI is ambiguous in patients with recent MI in whom biomarker levels have not have peaked at the time of the intervention, making it difficult to distinguish new PCI-related myonecrosis from the natural evolution of biomarker release from the presenting MI.^16^ Limiting this analysis to patients in whom CK-MB or cTnI levels were elevated but stable or falling before the PCI overcame this limitation. However, this requirement eliminated most patients undergoing true primary PCI, although the median duration from MI presentation to PCI was only 3 days.

In our LMCAD cohort with recent MI (at a median of 3 days from hospital presentation with MI to PCI), additional myonecrosis was common after PCI, more so when assessed by cTnI rather than by CK-MB (51.1% vs 27.5%). However, most levels of myocardial injury after PCI were not associated with subsequent 3-year mortality, even in this high-risk cohort. Neither the commonly used criteria for myocardial injury nor any level of cTnI elevation (even ≥70× URL) was prognostic.^17^^,^^18^ Most CK-MB elevations were also not related to outcomes, although an absolute incremental increase in CK-MB of ≥10× URL was a strong independent determinant of early and late mortality after PCI in this cohort. These findings are similar to but differ somewhat from those of the Evaluation of XIENCE versus Coronary Artery Bypass Surgery for Effectiveness of Left Main Revascularization (EXCEL) trial, in which only large biomarker elevations (not only peak CK-MB ≥10× URL but also cTn ≥70× URL) were associated with increased 5-year death after revascularization (both PCI and CABG) of LMCAD.^12^ The difference in the prognostic relationship after large cTn elevations between this study and EXCEL trial may be due to differences in the specific cTn assays used in each study or the fact that the EXCEL trial enrolled mostly patients with chronic coronary syndromes. Similarly, in the Synergy Between PCI With Taxus and Cardiac Surgery (SYNTAX) trial, in patients with 3-vessel and LMCAD, a prognostic relationship between CK-MB and 1-year and 10-year mortality was observed mainly with a peak CK-MB value of ≥10× URL.^11^ Thus, this study confirms and extends this understanding to an even potentially higher-risk LMCAD cohort with recent MI. Taken together with previous publications, it seems reasonable to use CK-MB as the preferred cardiac biomarker for detecting clinically relevant PMI after PCI for LMCAD in patients with recent MI; however, CK-MB is measured less frequently; is unavailable in most PCI centers and selective ascertainment of cTn assays, especially high-sensitivity assays; and for selective cases, has progressively become the preferred cardiac biomarker. In this regard, the threshold indicative of a clinically relevant event needs to be established if we are to switch exclusively to using cTn to define PMI.

The optimal definition of clinically relevant PMI after PCI (and CABG) remains a topic of considerable debate. In this study, PMI defined by 5 criteria (SCAI, ARC-2, fourth UDMI, and the ISCHEMIA primary and secondary definitions) was independently associated with 3-year CV and all-cause mortality. However, PMI defined according to the SCAI definition (which in most cases requires a CK-MB rise of ≥10× URL) was the strongest independent predictor of mortality. In addition, when the large SCAI PMIs were removed from the ARC-2 and fourth UDMI PMIs, the smaller ARC-2 and fourth UDMI PMIs that remained were no longer related with mortality. This finding supports the use of the SCAI criteria as the preferred definition for a clinically relevant PMI (especially if CK-MB is available). These observations are also consistent with those in other studies reporting that the SCAI definition of PMI has a stronger relationship with mortality than other PMI definitions in widespread use.^17^, ^18^, ^19^, ^20^ In contrast, in the SYNTAX trial, the prognostic effect of PMI after PCI as defined by the fourth UDMI was slightly greater than PMI defined by the ISCHEMIA primary and SCAI and EXCEL definitions.^11^ However, all PMIs in SYNTAX were derived from CK-MB measures, not the more sensitive cTn elevations that are preferred by UDMI and that represent substantially lesser degrees of myonecrosis.^17^^,^^21^^,^^22^

Presentation of LMCA disease with MI is not uncommon and represents a high-risk cohort for revascularization owing to frequent left ventricular dysfunction and the large amount of myocardium jeopardized. For these patients, any additional myonecrosis may be especially deleterious. The increased risk of all-cause mortality after PMI are a consequence of risk factors linked to the procedural complexity and/or the patient’s vulnerability and atherosclerosis burden or a result from the extent of cardiac injury. Our results showed higher aHRs for 3-year all-cause death with a higher post-PCI CK-MB threshold, confirming that large extents of periprocedural myocardial injury have a stronger prognostic effect in comparison with smaller extents of periprocedural myocardial injury. Of note, angiographic evidence of myocardial ischemia—a loss of branch or distal embolization—was present in approximately 60% of patients of our cohort with ARC-2 and fourth UDMI PMI definitions (Supplemental Table S15). Therefore, the long-term care of patients with PMI should be similar to that for patients with spontaneous MI and targeted for optimal secondary prevention based on the current guidelines.

### Limitations

As a post hoc analysis from a large real-world prospective registry, this study should be considered hypothesis generating. First, the data were derived from a high-volume single center with extensive LMCAD PCI operator experience,^13^^,^^23^ the generalizability of our observational study was limited. Second, as with all prior studies, death was the only outcome variable. Further studies are required to determine the effects of PMI on left ventricular function, heart failure, and quality of life. Third, although the largest study of its kind, the total number of PMIs and deaths are modest. The relatively small sample size presenting with recent MI undergoing PCI for LMCAD may have precluded detecting modest but significant relationships between smaller degrees of myonecrosis and death or interactions between subgroups, all of which limit the statistical power of this study. Fourth, patients with MI were excluded from this analysis if their cardiac biomarker levels were elevated and potentially rising before PCI at baseline; thus, our results do not apply to patients with MI in whom PCI is required before the elevated biomarkers were stable or falling for clinical instability. In this regard, the median time to PCI for LMCAD was 3 days (IQR, 1-8) in patients with recent MI. Furthermore, in patients with recent MI, it can be difficult to determine whether the left main lesion was the culprit, versus a non-LM lesion being the culprit with severe LM disease as a bystander. Fifth, although 99.7% of patients had serial CK-MB assessments, absent full government reimbursement cTnI measures were recommended but not mandated; hence, they were collected in only 62.6% of patients (albeit simultaneous with CK-MB, a strength of this study). Thus, we cannot exclude that some degree of selection bias (and loss of power) may have affected the cTnI findings. Sixth, our cTn findings are specific to the standard (nonhigh sensitivity) cTnI assay used at our center. Whether the results would have been different with cTnT or high-sensitivity cTn assays is unknown. Finally, although the patients were at high risk (LMCAD with recent MI), as reflected in the nearly 10% 3-year mortality rate, left ventricular function was preserved in most patients at baseline. We cannot exclude a greater effect of PMI (or of less extensive myonecrosis) in even higher-risk patients, although the principal findings were consistent in the 10% of patients with pulmonary edema and cardiogenic shock.

## Conclusions

Numerous studies have attempted to elucidate the optimal biomarker, threshold, and criteria to define a clinically relevant PMI after PCI (and CABG). As recently stated in a perspective on LMCAD revascularization, “The validation and widespread acceptance of a single harmonized definition of periprocedural MI is one of the key urgencies in cardiovascular research.”^24^ Among high-risk patients presenting with recent MI who underwent PCI for LMCAD (at a median of 3 days from hospital presentation with MI to PCI) in this study, only an absolute incremental post-PCI increase in CK-MB of ≥10× URL was independently predictive of 3-year CV and all-cause mortality, whereas even large elevations of cTnI were not. Because the prognostic effect of extensive myonecrosis as reflected in an incremental rise in CK-MB of ≥10× URL was not affected by the presence (or absence) of ancillary evidence of myocardial ischemia (ECG changes, angiographic complications of imaging loss of myocardium), an incremental CK-MB elevation of ≥10× URL is an attractive option to consider as a stand-alone measure to diagnose clinically relevant post-PCI myonecrosis. Among the PMI definitions in widespread use, the SCAI definition was most strongly related to early and late mortality, and the subset of SCAI PMI events largely explained the prognostic correlations from PMI definitions also incorporating lesser degrees of myonecrosis. These findings may inform prognostic guidance for clinical care pathways and the use and interpretation of end points in comparative randomized trials.

## Declaration of competing interest

Gregg W. Stone has received speaker honoraria from Medtronic, Pulnovo, and Infraredx; has served as a consultant to Valfix, TherOx, Robocath, HeartFlow, Ablative Solutions, Vectorious, Miracor, Neovasc, Abiomed, Ancora, Elucid Bio, Occlutech, CorFlow, Apollo Therapeutics, Impulse Dynamics, Cardiomech, Gore, Amgen, and Adona Medical; and has equity/options from Ancora, Cagent, Applied Therapeutics, Biostar family of funds, SpectraWave, Orchestra Biomed, Aria, Cardiac Success, Valfix, and Xenter. His daughter is an employee at Medtronic. His employer, Mount Sinai Hospital, receives research support from Abbott, Abiomed, Bioventrix, Cardiovascular Systems Inc, Phillips, Biosense-Webster, Shockwave, Vascular Dynamics, and V-wave. Hao-Yu Wang, Bo Xu, Kefei Dou, Changdong Guan, Lei Song, Yunfei Huang, Rui Zhang, Lihua Xie, Weixian Yang, Yongjian Wu, Shubin Qiao, Yuejin Yang, and Runlin Gao reported no financial interests.

## Funding sources

This study was supported by the National High Level Hospital Clinical Research Funding, Fuwai Hospital, Chinese Academy of Medical Sciences, and Peking Union Medical College (grant number 2022-GSP-GG-20).

## Ethics statement and patient consent

The research reported has adhered to the relevant ethical guidelines. The registry was approved by the Fuwai Hospital Institutional Review Board and complied with the tenets of the Declaration of Helsinki. All patients provided written informed consent
