# Supplementary material for: Effect of Periprocedural Myocardial Infarction After Initial Revascularization With Left Main PCI in Patients With Recent Myocardial Infarction
Source: J Soc Cardiovasc Angiogr Interv. 2023 Apr 3;2(3):100576. doi: 10.1016/j.jscai.2022.100576 (PMC11307394; doi:10.1016/j.jscai.2022.100576)
Supplement: Supplementary Appendix [file mmc1.docx]

**SUPPLEMENTARY APPENDIX**

**Impact of Periprocedural Myocardial Infarction After Initial Revascularization with Left Main PCI for Patients with Recent Myocardial Infarction**

| **Table of Contents** | | **Page Number** |
| --- | --- | --- |
| Supplementary Appendix 1 | Methods and Definitions | 3 |
| Supplementary Table 1 | Criteria for Commonly Used Definitions for Periprocedural Myocardial Injury in Patients with Recent ACS | 6 |
| Supplementary Table 2 | Criteria for Commonly Used Definitions for Periprocedural Myocardial Infarction in Patients with Recent ACS | 7 |
| Supplementary Table 3 | Frequency of Absolute Incremental Increase in CK-MB or cTnI from Baseline at Different Thresholds After Left Main PCI in Patients with Recent ACS | 10 |
| Supplementary Table 4 | Clinical Outcomes at 30 Days, 1 Year, and 3 Years (Cumulative) | 11 |
| Supplementary Table 5 | Overlap Between Periprocedural MI According to Different Definitions | 12 |
| Supplementary Table 6 | Impact of Periprocedural Myocardial Injury on 3-Year Cardiovascular and All-cause Mortality | 13 |
| Supplementary Table 7 | Sensitivity Analysis for Hazard Ratios for 3-year Cardiovascular and All-cause Mortality in Patients With versus Without Periprocedural MI | 14 |
| Supplementary Table 8 | Impact of Absolute Postprocedural cTnI Increase from Baseline With or Without Supporting Evidence of Myocardial Ischemia on 3-Year Cardiovascular and All-cause Mortality | 15 |
| Supplementary Table 9 | Impact of Absolute Postprocedural cTnI Increase from Baseline ≥5× URL With Each Supporting Evidence of Myocardial Ischemia on 3-Year Cardiovascular and All-cause Mortality | 17 |
| Supplementary Table 10 | Impact of Absolute Postprocedural CK-MB Increase from Baseline With or Without Supporting Evidence of Myocardial Ischemia on 3-Year Cardiovascular and All-cause Mortality | 18 |
| Supplementary Table 11 | Impact of Absolute Postprocedural CK-MB Increase from Baseline ≥10× URL With Each Supporting Evidence of Myocardial Ischemia on 3-Year Cardiovascular and All-cause Mortality | 20 |
| Supplementary Table 12 | 3-Year Outcomes According to Periprocedural Myocardial Infarction and the Presence of Recent ACS Within 24 Hours | 21 |
| Supplementary Table 13 | 3-Year Outcomes According to Periprocedural Myocardial Infarction and the Presence of Pulmonary Edema or Cardiogenic Shock | 22 |
| Supplementary Table 14 | Hazard Ratios for 3-year Cardiovascular and All-cause Mortality in Patients With versus Without Periprocedural Myocardial Infarction According to the ISCHEMIA Definition | 23 |
| Supplementary Table 15 | Supporting Imaging Evidence of New Myocardial Ischemia in Adjudicated PMI According to Different Definitions | 24 |
| Supplementary Figure 1 | Time-to-event Curves for 3-year Cardiovascular and All-cause Death According to Absolute Rise in Post-PCI CK-MB and cTn Levels from Baseline. | 25 |

**Supplementary Appendix 1. Methods and Definitions**

**Procedures**

PCI was performed with standard interventional techniques according to the guidelines valid at the time of presentation (1,2); stent choice and treatment strategy for stenting technique (including the approach to bifurcation stenting, pre-dilation and post-dilation, final kissing ballooning [FKB]) were based on operators’ preferences based on the angiographic and imaging findings. Route selection for catheterization, adjunctive devices and drugs to support PCI, and use of intravascular imaging were left to the operator’s discretion. The antithrombotic regimen (including its dose and duration) was based on parenteral heparin or bivalirudin, and dual antiplatelet therapy (DAPT) with oral aspirin plus a P2Y_12_ inhibitor according to the ischemic and bleeding risks for each individual patient’s clinical situation as well as treating physicians’ preference. Briefly, unfractionated heparin (initial bolus of 100 IU per kg body weight) or bivalirudin was administered during the procedure for procedural anticoagulation. Prior to PCI, patients routinely received loading doses of aspirin (300 mg) and a P2Y_12_ inhibitor (clopidogrel 600 mg or ticagrelor 180 mg). The majority of patients were prescribed clopidogrel because ticagrelor was approved in China in December 2013. After the procedure, patients were prescribed 100 mg/day aspirin indeﬁnitely and a P2Y_12_ inhibitor (clopidogrel 75 mg once daily or ticagrelor 90 mg twice daily) for 12 months. The routinely recommended duration of DAPT was 12 months, modified among patients taking oral anticoagulants (1,2). Medications such as statins, beta-blockers, and renin–angiotensin–aldosterone system blockers were prescribed as per the guidelines.

**Data Collection and Patient Follow-up**

Patients were systematically and prospectively followed at 1 month, 6 months, and 1 year and then annually through 3 years. At the time of the present analysis, all patients had completed 3 years of follow-up. Source data were solicited in case of potential endpoint-related adverse events. The follow-up data were collected during interviews conducted by trained clinical research coordinators by a health questionnaire with questions regarding vital status, rehospitalization, any adverse events, and status of antiplatelet therapy. If the patient did not visit the hospital, the outcome data were retrieved from hospital electronic medical records and telephone interviews or mail. For patients who underwent treatment for adverse events at other hospital, external medical records, discharge letters, and coronary angiography documentation were collected.

**Biomarker assessment**

Since 2004, blood samples for CK-MB have been routinely collected pre-PCI, every 8 hours for 24 hours post-PCI, and daily thereafter. In case of an increase in postprocedural CK-MB or cTnI or suspected myocardial ischemic events (eg, in the presence of new ischemic symptoms, ECG changes, or procedural complications), more frequent measurements were obtained every 6 to 8 hours to assess peak post-PCI CK-MB and cTnI levels. The immunosuppressive (Creatine Kinase MB Isozyme Assay Kit, BioSino Bio-Technology & Science) or chemiluminescent (Access CK-MB assay, Beckman Coulter-Access II Immunoassay System) assays were used to measure CK-MB levels. Since 2008, standard (non-high-sensitivity) cTnI levels (Access AccuTnI assay using chemiluminescent immunoassay method, Beckman Coulter-Access II Immunoassay System) were also drawn and analyzed in some patients according to available reimbursement. Baseline and peak CK-MB and cTnI levels within 48 hours post-PCI were normalized to the upper reference limit (URL). The URLs for the CK-MB and cTnI assays used were 24 IU/L (4 ng/mL) and 0.04 ng/mL, respectively. Compared with cardiac troponin (cTn), CK-MB is a simple, well-validated, standardized, inexpensive laboratory marker that was reimbursed in our country at the time of the studies; therefore, 99.7% (n = 349) of patients had serial CK-MB assessment in our hospital. Given cost considerations for patients and absent full reimbursement by the government for this test, standard (non-high sensitivity) cTnI values were not routinely collected in all patients. Post-procedural cTnI was recommended (but not required) to be simultaneously drawn with CK-MB if possible. As a result, cTnI values were collected in approximately 62.6% of patients in whom PCI was performed.

**Selected endpoint definitions**

Non-procedural myocardial infarction (MI) was defined according to the Third Universal MI definition during adjudication (3).

Any one of the following criteria meets the diagnosis of non-procedural MI:

- Spontaneous MI (> 48 hours after PCI): Detection of rise and/or fall of cardiac biomarkers (preferably troponin) with at least one value above the upper reference limit (URL) together with evidence of myocardial ischemia with at least one of the following:

1. Symptoms of acute myocardial ischemia
2. ECG changes indicative of new ischemia [new significant ST-segment elevation or depression, or new left bundle branch block (LBBB)]
3. Development of pathological Q waves (≥0.04 seconds in duration and ≥1 mm in depth) in ≥2 contiguous precordial leads or ≥2 adjacent limb leads) of the ECG
4. Identification of an intracoronary thrombus by angiography or autopsy
5. Imaging evidence of new loss of viable myocardium or new regional wall motion abnormality

- Cardiac death with symptoms suggestive of MI and presumed new ischemic ECG changes or new LBBB, but death occurred before cardiac biomarkers were obtained, or before cardiac biomarker values would be increased.
- Stent thrombosis associated with MI when detected by coronary angiography or autopsy in the setting of myocardial ischemia and with a rise and/or fall of cardiac biomarker values with at least one value above the 99th percentile URL

Stent thrombosis was defined as the occurrence of definite or probable stent thrombosis according to the Academic Research Consortium Definition (4).

Definite stent thrombosis was considered to have occurred by either angiographic or pathological confirmation.

1. Angiographic confirmation of stent thrombosis: the presence of thrombus that originates in the stent or in the segment 5 mm proximal or distal to the stent and presence of at least 1 of the following criteria within a 48-hour window:
2. Acute ischemic symptoms at rest
3. New ischemic ECG changes that suggest acute ischemia
4. Typical rise and fall in cardiac biomarkers that represent a spontaneous MI
5. Non-occlusive thrombus: Intracoronary thrombus defined as a (spheric, ovoid, or irregular) noncalcified filling defect or lucency surrounded by contrast material (on 3 sides or within a coronary stenosis) seen in multiple projections, or persistence of contrast material within the lumen, or visible embolization of intraluminal material downstream.
6. Occlusive thrombus: TIMI 0 or TIMI 1 intrastent or proximal to a stent up to the most adjacent proximal side branch or main branch (if originates from the side branch)
7. Pathological confirmation of stent thrombosis: Evidence of recent thrombus within the stent determined at autopsy or via examination of tissue retrieved following thrombectomy.

Probable stent thrombosis was considered to have occurred after intracoronary stenting in the following cases:

1. Any unexplained death within the first 30 days following PCI
2. Any MI at any time following PCI that is related to documented acute ischemia in the territory of the implanted stent, in the absence of angiographic/pathological confirmation of stent thrombosis and no other obvious cause

**References**

1. Wijns W, Kolh P, Danchin N et al. Guidelines on myocardial revascularization. Eur Heart J 2010;31:2501-55.

2. Windecker S, Kolh P, Alfonso F et al. 2014 ESC/EACTS Guidelines on myocardial revascularization: The Task Force on Myocardial Revascularization of the European Society of Cardiology (ESC) and the European Association for Cardio-Thoracic Surgery (EACTS)Developed with the special contribution of the European Association of Percutaneous Cardiovascular Interventions (EAPCI). Eur Heart J 2014;35:2541-619.

3. Thygesen K, Alpert JS, Jaffe AS et al. Third universal definition of myocardial infarction. J Am Coll Cardiol 2012;60:1581-98.

4. Cutlip DE, Windecker S, Mehran R et al. Clinical end points in coronary stent trials: a case for standardized definitions. Circulation 2007;115:2344-51.

**Supplementary Table 1. Criteria for Commonly Used Definitions for Periprocedural Myocardial Injury in Patients with Recent ACS**

|  | **Definitions*** |
| --- | --- |
| **Periprocedural myocardial injury**  **(based on Fourth UDMI criteria) ^(5)^** | Absolute rise in cTn from baseline >1× URL or a rise of cTn values >20% of the baseline value (used preferentially if available), or absolute rise in CK-MB from baseline >1× URL or a rise of CK-MB values >20% of the baseline value |
| **Significant periprocedural myocardial injury**  **(based on ARC-2 criteria) ^(6)^** | Absolute rise in cTn from baseline ≥70× URL (used preferentially if available), or absolute rise in CK-MB from baseline ≥10× URL |

*Periprocedural myocardial injury was defined in patients in whom baseline CK-MB or cTn levels was elevated and stable or falling.

**References**

5. Thygesen K, Alpert JS, Jaffe AS, et al. Fourth Universal Definition of Myocardial Infarction (2018). Circulation. 2018 Nov 13;138(20):e618-e651.

6. Garcia-Garcia HM, McFadden EP, Farb A, et al. Standardized End Point Definitions for Coronary Intervention Trials: The Academic Research Consortium-2 Consensus Document. Eur Heart J. 2018 Jun 14;39(23):2192-2207.

**Supplementary Table 2. Criteria for Commonly Used Definitions for Periprocedural Myocardial Infarction in Patients with Recent ACS**

| **Definition*** | **Time after procedure** | **Peak biomarker threshold** | **Supporting evidence required** |
| --- | --- | --- | --- |
| **SCAI** | Within 48 hrs | In patients with elevated pre-PCI biomarker levels that are stable or falling:  i) An absolute rise in CK-MB from baseline ≥10× URL (used preferentially if available) or absolute increase in cTn from baseline ≥70× URL), or  ii) An absolute rise in CK-MB ≥5×URL (used preferentially if available) or absolute increase in cTn from baseline ≥35× URL | i) None  ii) ECG: New pathological Q waves in ≥2 contiguous leads or new persistent LBBB |
| **ARC-2** | Within 48 hrs | In patients with elevated pre-PCI biomarker levels that are stable or falling:  An absolute rise in cTn from baseline ≥35× URL (used preferentially if available), or an absolute rise in CK-MB from baseline ≥5× URL | *One or more of the following:*  ECG: New significant Q waves or equivalent  Angiographic: Occlusion of a major epicardial artery or a side branch, major coronary dissection, disruption of collateral flow, distal embolization, or persistent slow flow or no reflow  Imaging: New substantial loss of myocardium on imaging |
| **4^th^ UDMI** | Within 48 hrs | In patients with elevated pre-PCI biomarker levels that are stable or falling:  cTn rise by >20% from baseline and the absolute cTn value must still be >5×URL (used preferentially if available), or CK-MB rise by >20% from baseline and the absolute CK-MB value must still be >5×URL | *One or more of the following:*  ECG: New ischemic ECG changes (ST segments) or development of new pathological Q waves  Angiographic: Occlusion of a major epicardial artery or a side branch, major coronary dissection, disruption of collateral flow, distal embolization, or persistent slow flow or no reflow  Imaging: Evidence of new loss of viable myocardium or new regional wall motion abnormality in a pattern consistent with an ischemic etiology |
| **ISCHEMIA**   - **Primary definition** | Within 48 hrs | In patients with elevated pre-PCI biomarker levels that are stable or falling:  CK-MB rise (used preferentially if available) by >20% from baseline and the absolute CK-MB value must still be >5×URL, or cTn rise (if CK-MB is unavailable) by >20% from baseline and the absolute cTn value must still be >35×URL | *One or more of the following:*  ECG: New ECG changes (ST segment elevation or depression >0.1mV in 2 contiguous leads), new pathologic Q-waves in ≥2 contiguous leads, or new persistent LBBB present on a post-PCI ECG obtained at least 30 minutes and up to 48 hours post procedure in the absence of any intervening coronary event between the time of the PCI procedure and the ECG showing changes.  Angiographic: Post-procedure angiographic TIMI 0/1 flow in a major coronary artery or a side branch with reference vessel diameter ≥2.0 mm which had TIMI 2-3 flow at baseline, or TIMI 2 flow in a major coronary artery or a side branch with reference vessel diameter ≥3.0 mm which had TIMI 3 flow at baseline or Type C dissection (NHLBI classification) or greater in the target vessel |
| - **Secondary definition** | Within 48 hrs | In patients with elevated pre-PCI biomarker levels that are stable or falling:  cTn rise (used preferentially if available) by >20% from baseline and the absolute cTn value must still be >5×URL, or CK-MB rise (if cTn is unavailable) by >20% from baseline and the absolute CK-MB value must still be >5×URL | *One or more of the following:*  Symptoms: Symptoms suggestive of myocardial ischemia (≥20 min)  ECG: New ischemic ST changes or new pathological Q waves.  Angiographic: Angiographic evidence of a flow limiting complication, such as loss of patency of a side branch, persistent slow-flow or no re-flow, embolization, or Type C dissection (NHLBI classification) or greater in the target vessel.  Imaging: Imaging evidence of new loss of viable myocardium or new regional wall motion abnormality. |

*For the SCAI and ISCHEMIA primary definitions CK-MB is used preferentially if available, cTn otherwise. For the ARC-2, 4^th^ UDMI, and ISCHEMIA secondary definitions cTn is used preferentially if available, CK-MB otherwise. In the Fu Wai angiographic core laboratory, a major side branch occlusion was deﬁned as a branch supplying the left ventricle (including diagonal, posterolateral, posterior descending, obtuse marginal, and septal branches) that was ≥1.5 mm in diameter; major coronary dissection was defined as dissection in the target vessel greater than type B from National Heart, Lung, and Blood Institute classification; disruption of collateral flow was defined as reduction in collateral flow by ≥1 grades (Rentrop classification); distal embolization was defined as the appearance of an abrupt cutoff in the distal vessel (or in a side branch ≥1.5 mm) after PCI; persistent slow flow or no reflow was defined as markedly delayed flow (TIMI grade 2 for slow flow, TIMI 0 or 1 for no reflow) in a target vessel with minimal (<30%) residual stenosis at the stented/scaffolded segment and no evidence of flow-limiting dissection.

CKMB = creatine kinase MB band. cTn = cardiac troponin. ECG = Electrocardiographic. LBBB = left bundle branch block. PCI = percutaneous coronary intervention. URL = upper reference limit.

**Supplementary Table 3. Frequency of** **Absolute Incremental Increase in CK-MB or cTnI from Baseline at Different Thresholds After Left Main PCI in Patients with Recent ACS**

| **Threshold** | **Absolute increase in CK-MB**  **(n = 349)** | **Absolute increase in cTnI**  **(n = 219)** |
| --- | --- | --- |
| No elevation | 72.5% (253) | 48.9% (107) |
| ≥1×URL | 27.5% (96) | 51.1% (112) |
| ≥3×URL | 15.5% (54) | 43.4% (95) |
| ≥5×URL | 12.0% (42) | 37.4% (82) |
| ≥10×URL | 6.3% (22) | 32.0% (70) |
| ≥35×URL | 0.9% (3) | 16.0% (35) |
| ≥70×URL | 0.6% (2) | 11.4% (25) |

Values are % (n). CK-MB = creatine kinase-MB; cTn = cardiac troponin; URL = upper reference limit.

**Supplementary Table 4. Clinical Outcomes at 30 Days, 1 Year, and 3 Years (Cumulative)**

| **Outcome** | **30 days** | **1 year** | **3 years** |
| --- | --- | --- | --- |
| All-cause death | 3.4% (12) | 6.3% (22) | 9.8% (34) |
| Cardiovascular death | 3.4% (12) | 5.7% (20) | 7.2% (25) |
| Periprocedural myocardial infarction |  |  |  |
| SCAI | 8.6% (30) | - | - |
| ARC-2 | 12.3% (43) | - | - |
| 4^th^ UDMI | 19.4% (68) | - | - |
| Non-procedural myocardial infarction | 1.2% (4) | 3.5% (12) | 5.3% (18) |
| Any revascularization | 10.6% (5) | 8.1% (27) | 10.6% (35) |
| Target vessel revascularization | 0.3% (1) | 4.6% (15) | 6.3% (20) |
| Target lesion revascularization | 0.3% (1) | 3.7% (12) | 4.7% (15) |
| Deﬁnite or probable stent thrombosis | 0.3% (4) | 2.3% (8) | 4.6% (12) |
| Stroke | 0% (0) | 0% (0) | 1.3% (5) |

Values are Kaplan-Meier estimates (number of events). ARC-2 = Academic Research Consortium-2; SCAI = Society for Cardiovascular Angiography and Interventions; UDMI = Universal Definition of Myocardial Infarction.

30 days, 1-year, and 3-year follow-up data were available in 350 of 350 patients (100%), 350 of 350 patients (100%), and 346 of 350 patients (98.9%), respectively.

12, 22, and 34 patients were died within 30 days, 1 year, and 3 years; therefore, 338, 328, and 316 patients were evaluated at 30 days,1 year, and 3 years.

**Supplementary Table 5. Overlap Between Periprocedural Myocardial Infarction According to Different Definitions**

|  | **N** |
| --- | --- |
| **4^th^ UDMI** | 68 |
| 4^th^ UDMI and ARC-2 | 43 |
| 4^th^ UDMI and SCAI | 25 |
| 4^th^ UDMI and ARC-2 and SCAI | 24 |
| 4^th^ UDMI only (excluding SCAI and ARC-2) | 24 |
| **ARC-2** | 43 |
| ARC-2 and SCAI | 24 |
| ARC-2 only (excluding SCAI) | 19 |
| **SCAI** | 30 |

ARC-2 = Academic Research Consortium-2; SCAI = Society for Cardiovascular Angiography and Interventions; 4^th^ UDMI = Fourth Universal Definition of Myocardial Infarction.

**Supplementary Table 6. Impact of Periprocedural Myocardial Injury on 3-Year Cardiovascular and All-cause Mortality**

| **Biomarker Threshold** | **% (n/N)** | **Unadjusted HR**  **(95% CI)** | ***P* Value** | **Adjusted HR***  **(95% CI)** | ***P* Value** |
| --- | --- | --- | --- | --- | --- |
| **Cardiovascular mortality** | | | | | |
| Periprocedural myocardial injury (Fourth UDMI) | 5.8% (9/155) | 0.70 (0.31-1.59) | 0.397 | 0.66 (0.29-1.53) | 0.333 |
| Significant periprocedural myocardial injury (ARC-2) | 12.1% (4/33) | 1.94 (0.66-5.64) | 0.226 | 1.94 (0.65-5.82) | 0.236 |
| **All-cause mortality** | | | | | |
| Periprocedural myocardial injury (Fourth UDMI) | 7.7% (12/155) | 0.68 (0.34-1.37) | 0.281 | 0.65 (0.31-1.32) | 0.232 |
| Significant periprocedural myocardial injury (ARC-2) | 12.1% (4/33) | 1.36 (0.48-3.85) | 0.567 | 1.31 (0.45-3.82) | 0.616 |

Event rates are expressed as Kaplan-Meier estimated rates (%) at 3 years (number of events/denominator at baseline). Adjusted hazard ratios and 95% confidence intervals were generated using multivariable Cox regression analysis. *Model adjusted for age, sex, current smoking, hypertension, diabetes mellitus, prior myocardial infarction, and 2- or 3-vessel disease. CI = conﬁdence interval; CK-MB = creatine kinase-MB; HR= hazard ratio; URL = upper reference limit.

**Supplementary Table 7. Sensitivity Analysis for Hazard Ratios for 3-year Cardiovascular and All-cause Mortality in Patients With versus Without Periprocedural MI**

|  | **Patients with**  **periprocedural MI** | **Patients without**  **periprocedural MI** | **Unadjusted HR**  **(95% CI)** | ***P* Value** |
| --- | --- | --- | --- | --- |
|  |  |  |  |  |
| **Cardiovascular Mortality** | | | | |
| ARC-2 PMIs excluding SCAI PMIs | 5.3% (1/19) | 7.3% (24/331) | 0.71 (0.10-5.28) | 0.74 |
| 4^th^ UDMI PMIs excluding SCAI or ARC-2 PMIs | 8.3% (2/24) | 7.1% (23/326) | 1.22 (0.29-5.16) | 0.79 |
| **All-cause Mortality** | | | | |
| ARC-2 PMIs excluding SCAI PMIs | 10.5% (2/19) | 9.3% (32/331) | 1.08 (0.26-4.48) | 0.92 |
| 4^th^ UDMI PMIs excluding SCAI or ARC-2 PMIs | 12.5% (3/24) | 9.5% (31/326) | 1.37 (0.42-4.48) | 0.60 |

Event rates are expressed as Kaplan-Meier estimated rates (%) at 3 years (number of events/denominator at baseline). AMI = acute myocardial infarction; ARC-2 = Academic Research Consortium-2; PMI = periprocedural myocardial infarction; SCAI = Society for Cardiovascular Angiography and Interventions; 4^th^ UDMI = Fourth Universal Definition of Myocardial Infarction.

**Supplementary Table 8. Impact of Absolute Postprocedural cTnI Increase from Baseline With or Without Supporting Evidence of Myocardial Ischemia on 3-Year Cardiovascular and All-cause Mortality**

| **Biomarker Threshold** | **% (n/N)** | **Unadjusted HR**  **(95% CI)** | ***P* Value** | **Adjusted HR***  **(95% CI)** | ***P* Value** |
| --- | --- | --- | --- | --- | --- |
| **Cardiovascular Mortality** | | | | | |
| **△ (peak-baseline) cTnI ≥5× URL** | 8.5% (7/82) | 4.17 (1.08-16.13) | 0.039 | 3.56 (0.90-14.16) | 0.07 |
| With supporting evidence of myocardial ischemia† | 10.4% (5/49) | 3.68 (1.07-12.72) | 0.039 | 3.09 (0.85-11.21) | 0.09 |
| Without supporting evidence of myocardial ischemia† | 6.1% (2/33) | 1.47 (0.31-6.93) | 0.63 | 1.42 (0.29-7.06) | 0.67 |
| **△ (peak-baseline) cTnI ≥35× URL** | 8.6% (3/35) | 2.28 (0.59-8.81) | 0.23 | 1.93 (0.46-8.18) | 0.37 |
| With supporting evidence of myocardial ischemia† | 11.1% (3/27) | 3.14 (0.81-12.12) | 0.10 | 2.98 (0.72-12.33) | 0.13 |
| Without supporting evidence of myocardial ischemia† | 0% (0/8) | - | - | - | - |
| **All-cause Mortality** | | | | | |
| **△ (peak-baseline) cTnI ≥5× URL** | 14.6% (12/82) | 3.62 (1.36-9.64) | 0.01 | 3.13 (1.16-8.47) | 0.19 |
| With supporting evidence of myocardial ischemia† | 14.5% (7/49) | 2.37 (0.92-6.11) | 0.08 | 1.93 (0.73-5.14) | 0.19 |
| Without supporting evidence of myocardial ischemia† | 15.2% (5/33) | 2.28 (0.81-6.40) | 0.12 | 2.36 (0.82-6.83) | 0.11 |
| **△ (peak-baseline) cTnI ≥35× URL** | 11.4% (4/35) | 1.52 (0.50-4.62) | 0.46 | 1.36 (0.42-4.38) | 0.61 |
| With supporting evidence of myocardial ischemia† | 14.8% (4/27) | 2.10 (0.69-6.38) | 0.19 | 2.05 (0.65-6.44) | 0.22 |
| Without supporting evidence of myocardial ischemia† | 0% (0/8) | - | - | - | - |

Event rates are expressed as Kaplan-Meier estimated rates (%) at 3 years (number of events/denominator at baseline). Adjusted hazard ratios and 95% confidence intervals were generated using multivariable Cox regression analysis. †Supporting electrocardiographic, angiographic, or imaging evidence of myocardial ischemia. *Model adjusted for age, sex, current smoking, hypertension, diabetes mellitus, prior myocardial infarction, and 2- or 3-vessel disease. CI = confidence interval; CK-MB = creatine kinase-MB; HR= hazard ratio; URL = upper reference limit.

**Supplementary Table 9. Impact of Absolute Postprocedural cTnI Increase from baseline ≥5× URL With Each Supporting Evidence of Myocardial Ischemia on 3-Year Cardiovascular and All-cause Mortality**

| **△ (peak-baseline) cTnI** | **cTnI Increase ≥5× URL with supporting evidence of new myocardial ischemia** | **Others** | **Unadjusted HR**  **(95% CI)** | ***P* Value** | **Adjusted HR***  **(95% CI)** | ***P* Value** |
| --- | --- | --- | --- | --- | --- | --- |
| **Cardiovascular Mortality** | | | | | | |
| cTnI increase ≥5× URL with ECG evidence | 8.6% (3/35) | 3.8% (7/184) | 2.35 (0.61-9.08) | 0.22 | 2.33 (0.57-9.56) | 0.24 |
| cTnI increase ≥5× URL with angiographic evidence | 6.5% (2/31) | 4.3% (8/188) | 1.56 (0.33-7.35) | 0.57 | 1.12 (0.22-5.75) | 0.72 |
| cTnI increase ≥5× URL with imaging evidence | 14.3% (1/7) | 4.2% (9/212) | 3.66 (0.46-28.88) | 0.22 | 2.94 (0.26-33.75) | 0.39 |
| **All-cause Mortality** | | | | | | |
| cTnI increase ≥5× URL with ECG evidence | 11.4% (4/35) | 7.6% (14/184) | 1.58 (0.52-4.80) | 0.42 | 1.61 (0.52-5.03) | 0.41 |
| cTnI increase ≥5× URL with angiographic evidence | 12.9% (4/31) | 7.4% (14/188) | 1.81 (0.59-5.49) | 0.30 | 1.37 (0.42-4.42) | 0.60 |
| cTnI increase ≥5× URL with imaging evidence | 14.3% (1/7) | 8.0% (17/212) | 1.95 (0.26-14.62) | 0.52 | 1.77 (0.19-16.17) | 0.61 |

Event rates are expressed as Kaplan-Meier estimated rates (%) at 3 years (number of events/denominator at baseline). Adjusted hazard ratios and 95% confidence intervals were generated using multivariable Cox regression analysis. *Model adjusted for age, sex, current smoking, hypertension, diabetes mellitus, prior myocardial infarction, and 2- or 3-vessel disease. CI = conﬁdence interval; cTn = cardiac troponin; ECG = electrocardiogram; HR= hazard ratio; URL = upper reference limit.

**Supplementary Table 10. Impact of Absolute Postprocedural CK-MB Increase from Baseline With or Without Supporting Evidence of Myocardial Ischemia on 3-Year Cardiovascular and All-cause Mortality**

| **Biomarker Threshold** | **% (n/N)** | **Unadjusted HR**  **(95% CI)** | ***P* Value** | **Adjusted HR***  **(95% CI)** | ***P* Value** |
| --- | --- | --- | --- | --- | --- |
| **Cardiovascular Mortality** | | | | | |
| **△ (peak-baseline) CK-MB ≥5× URL** | 23.8% (10/42) | 5.39 (2.42-12.00) | <0.0001 | 5.60 (2.50-12.58) | <0.0001 |
| With supporting evidence of myocardial ischemia† | 22.9% (8/35) | 4.62 (1.99-10.71) | 0.0004 | 5.00 (2.14-11.67) | 0.0002 |
| Without supporting evidence of myocardial ischemia† | 28.6% (2/7) | 4.84 (1.14-20.54) | 0.033 | 4.13 (0.94-18.17) | 0.06 |
| **△ (peak-baseline) CK-MB ≥10× URL** | 40.9% (9/35) | 10.33 (4.56-23.43) | <0.0001 | 11.47 (4.90-26.82) | <0.0001 |
| With supporting evidence of myocardial ischemia† | 38.9% (7/27) | 8.63 (3.60-20.68) | <0.0001 | 9.02 (3.68-22.13) | <0.0001 |
| Without supporting evidence of myocardial ischemia† | 50.4% (2/8) | 9.99 (2.35-42.54) | 0.002 | 12.13 (2.65-55.51) | 0.001 |
| **All-cause Mortality** | | | | | |
| **△ (peak-baseline) CK-MB ≥5× URL** | 23.8% (10/35) | 3.41 (1.63-7.14) | 0.001 | 3.65 (1.73-7.70) | 0.001 |
| With supporting evidence of myocardial ischemia† | 22.9% (8/35) | 3.05 (1.38-6.74) | 0.006 | 3.33 (1.50-7.40) | 0.003 |
| Without supporting evidence of myocardial ischemia† | 28.6% (2/7) | 3.57 (0.86-14.92) | 0.08 | 3.37 (0.78-14.47) | 0.103 |
| **△ (peak-baseline) CK-MB ≥10× URL** | 40.9% (9/22) | 6.80 (3.17-14.59) | <0.0001 | 7.77 (3.51-17.19) | <0.0001 |
| With supporting evidence of myocardial ischemia† | 38.9% (7/18) | 5.86 (2.56-13.50) | 0.006 | 6.22 (2.64-14.63) | <0.0001 |
| Without supporting evidence of myocardial ischemia† | 50.0% (2/4) | 9.99 (2.35-42.54) | 0.002 | 11.17 (2.47-50.47) | 0.002 |

Event rates are expressed as Kaplan-Meier estimated rates (%) at 3 years (number of events/denominator at baseline). Adjusted hazard ratios and 95% confidence intervals were generated using multivariable Cox regression analysis. †Supporting electrocardiographic, angiographic, or imaging evidence of myocardial ischemia. *Model adjusted for age, sex, current smoking, hypertension, diabetes mellitus, prior myocardial infarction, and 2- or 3-vessel disease. CI = conﬁdence interval; CK-MB = creatine kinase-MB; HR= hazard ratio; URL = upper reference limit.

**Supplementary Table 11. Impact of Absolute Postprocedural CK-MB Increase from baseline ≥10× URL With Each Supporting Evidence of Myocardial Ischemia on 3-Year Cardiovascular and All-cause Mortality**

| **△ (peak-baseline) CK-MB** | **CK-MB Increase ≥10× URL with supporting evidence of new myocardial ischemia** | **Others** | **Unadjusted HR**  **(95% CI)** | ***P* Value** | **Adjusted HR***  **(95% CI)** | ***P* Value** |
| --- | --- | --- | --- | --- | --- | --- |
| **Cardiovascular Mortality** | | | | | | |
| CK-MB increase ≥10× URL with ECG evidence | 35.3% (6/17) | 5.7% (19/332) | 7.07 (2.82-17.73) | <0.0001 | 7.34 (2.86-18.81) | <0.0001 |
| CK-MB increase ≥10× URL with angiographic evidence | 22.2% (2/9) | 6.8% (23/340) | 3.51 (0.83-14.89) | 0.09 | 4.62 (1.04-20.62) | 0.045 |
| CK-MB increase ≥10× URL with imaging evidence | 66.7% (2/3) | 6.6% (23/346) | 13.02 (3.06-55.41) | 0.001 | 9.62 (2.01-46.06) | 0.005 |
| **All-cause Mortality** | | | | | | |
| CK-MB increase ≥10× URL with ECG evidence | 35.3% (6/17) | 8.4% (28/332) | 4.89 (2.02-11.82) | 0.0004 | 5.15 (2.08-12.74) | 0.0004 |
| CK-MB increase ≥10× URL with angiographic evidence | 22.2% (2/9) | 9.4% (32/340) | 2.51 (0.60-10.48) | 0.21 | 2.87 (0.67-12.32) | 0.16 |
| CK-MB increase ≥10× URL with imaging evidence | 66.7% (2/3) | 9.2% (32/346) | 9.66 (2.31-40.37) | 0.002 | 6.63 (1.43-30.76) | 0.016 |

Event rates are expressed as Kaplan-Meier estimated rates (%) at 3 years (number of events/denominator at baseline). Adjusted hazard ratios and 95% confidence intervals were generated using multivariable Cox regression analysis. *Model adjusted for age, sex, current smoking, hypertension, diabetes mellitus, prior myocardial infarction, and 2- or 3-vessel disease. CK-MB = creatine kinase-MB; CI = conﬁdence interval; ECG = electrocardiogram; HR= hazard ratio; URL = upper reference limit.

**Supplementary Table 12. 3-Year Outcomes According to Periprocedural Myocardial Infarction and the Presence of MI within 24 Hours**

|  | **Timing of AMI >24 hours (n=226)** | | | **Timing of AMI ≤24 hours (n=124)** | | |  |
| --- | --- | --- | --- | --- | --- | --- | --- |
|  | **Patients with**  **periprocedural MI** | **Patients without**  **periprocedural MI** | **Unadjusted HR (95% CI)** | **Patients with**  **periprocedural MI** | **Patients without**  **periprocedural MI** | **Unadjusted HR (95% CI)** | ***P* for interaction** |
| **Cardiovascular death** | | | | | | | |
| SCAI definition | 12.5% (1/8) | 3.7% (8/218) | 3.60 (0.45-28.81) | 40.9% (9/22) | 6.9% (7/102) | 7.32 (2.72-19.71) | 0.540 |
| ARC-2 definition | 18.2% (2/11) | 3.3% (7/215) | 5.85 (1.22-28.20) | 21.9% (7/32) | 9.8% (9/92) | 2.40 (0.89-6.45) | 0.342 |
| 4^th^ UDMI deﬁnition | 13.8% (4/29) | 2.5% (5/197) | 5.70 (1.53-21.23) | 17.9% (7/39) | 10.6% (9/85) | 1.77 (0.66-4.74) | 0.156 |
| **All-cause death** | | | | | | | |
| SCAI definition | 12.5% (1/8) | 5.5% (12/218) | 2.44 (0.32-18.74) | 40.9% (9/22) | 11.8% (12/102) | 4.37 (1.84-10.39) | 0.594 |
| ARC-2 definition | 18.2% (2/11) | 5.1% (11/215) | 3.83 (0.85-17.26) | 25.0% (8/32) | 14.1% (13/92) | 1.92 (0.79-4.62) | 0.435 |
| 4^th^ UDMI deﬁnition | 17.2% (5/29) | 4.1% (8/197) | 4.64 (1.52-14.20) | 20.5% (8/39) | 15.3% (13/85) | 1.40 (0.58-3.39) | 0.099 |

Event rates are expressed as Kaplan-Meier estimated rates (%) at 3 years (number of events/denominator at baseline). AMI = acute myocardial infarction; ARC-2 = Academic Research Consortium-2; MI = myocardial infarction; PMI = Periprocedural myocardial infarction; SCAI = Society for Cardiovascular Angiography and Interventions; 4^th^ UDMI = Fourth Universal Definition of MI.

**Supplementary Table 13. 3-Year Outcomes According to Periprocedural Myocardial Infarction and the Presence of Pulmonary Edema or Cardiogenic Shock**

|  | **Without Pulmonary Edema or Cardiogenic Shock (n=318)** | | | **Pulmonary Edema or Cardiogenic Shock (n=32)** | | |  |
| --- | --- | --- | --- | --- | --- | --- | --- |
|  | **Patients with**  **periprocedural MI** | **Patients without**  **periprocedural MI** | **Unadjusted HR (95% CI)** | **Patients with**  **periprocedural MI** | **Patients without**  **periprocedural MI** | **Unadjusted HR (95% CI)** | ***P* for interaction** |
| **Cardiovascular death** | | | | | | | |
| SCAI definition | 11.8% (2/17) | 2.7% (8/301) | 4.58 (0.97-21.55) | 61.5% (8/13) | 36.8% (7/19) | 1.79 (0.65-4.96) | 0.364 |
| ARC-2 definition | 9.7% (3/31) | 2.4% (7/287) | 4.11 (1.06-15.88) | 50.0% (6/12) | 45.0% (9/20) | 1.06 (0.38-2.97) | 0.127 |
| 4^th^ UDMI deﬁnition | 7.3% (4/55) | 2.3% (6/263) | 3.33 (0.94-11.80) | 53.8% (7/13) | 42.1% (8/19) | 1.27 (0.46-3.52) | 0.277 |
| **All-cause death** | | | | | | | |
| SCAI definition | 11.8% (2/17) | 5.3% (16/301) | 2.29 (0.53-9.96) | 61.5% (8/13) | 42.1% (8/19) | 1.59 (0.59-4.26) | 0.774 |
| ARC-2 definition | 12.9% (4/31) | 4.9% (14/287) | 2.75 (0.91-8.36) | 50.0% (6/12) | 50.0% (10/20) | 0.95 (0.35-2.62) | 0.180 |
| 4^th^ UDMI deﬁnition | 10.9% (6/55) | 4.6% (12/263) | 2.51 (0.94-6.69) | 53.8% (7/13) | 47.4% (9/19) | 1.14 (0.42-3.06) | 0.309 |

Event rates are expressed as Kaplan-Meier estimated rates (%) at 3 years (number of events/denominator at baseline). AMI = acute myocardial infarction; ARC-2 = Academic Research Consortium-2; MI = myocardial infarction; PMI = Periprocedural myocardial infarction; SCAI = Society for Cardiovascular Angiography and Interventions; 4^th^ UDMI = Fourth Universal Definition of MI.

**Supplementary Table 14. Hazard Ratios for 3-year Cardiovascular and All-cause Mortality in Patients With versus Without Periprocedural Myocardial Infarction According to the ISCHEMIA Definition**

|  | **Patients with**  **periprocedural MI** | **Patients without**  **periprocedural MI** | **Unadjusted HR**  **(95% CI)** | ***P* Value** | **Adjusted HR***  **(95% CI)** | ***P* Value** |
| --- | --- | --- | --- | --- | --- | --- |
|  |  |  |  |  |  |  |
| **Cardiovascular Mortality** | | | | | | |
| ISCHEMIA primary PMI definition | 25.6% (10/39) | 4.8% (15/311) | 5.95 (2.67-13.25) | <0.001 | 6.55 (2.92-14.72) | <0.001 |
| ISCHEMIA secondary PMI definition | 16.7% (12/72) | 4.7% (13/278) | 3.84 (1.75-8.41) | 0.001 | 3.56 (1.61-7.86) | 0.002 |
| **All-cause Mortality** | | | | | | |
| ISCHEMIA primary PMI definition | 25.6% (10/39) | 7.7% (24/311) | 3.78 (1.81-7.90) | <0.001 | 4.25 (2.01-8.97) | <0.001 |
| ISCHEMIA secondary PMI definition | 19.4% (14/72) | 7.2% (14/278) | 2.95 (1.49-5.84) | 0.002 | 2.68 (1.34-5.34) | 0.005 |

Event rates are expressed as Kaplan-Meier estimated rates (%) at 3 years (number of events/denominator at baseline). Adjusted hazard ratios and 95% confidence intervals were generated using multivariable Cox regression analysis. *Model adjusted for age, sex, current smoking, hypertension, diabetes mellitus, prior myocardial infarction, and 2- or 3-vessel disease. ISCHEMIA = International Study of Comparative Health Effectiveness with Medical and Invasive Approaches; PMI = periprocedural myocardial infarction.

**Supplementary Table 15. Supporting Evidence of New Myocardial Ischemia in Adjudicated PMI According to Different Definitions**

| **Evidence of myocardial ischemia** | **ARC-2 PMI**  **(N=43)** | **4^th^UDMI PMI**  **(N=68)** |
| --- | --- | --- |
| New ischemic ECG changes | 25 (58.1%) | 35 (51.5%) |
| Development of new pathological Q waves | 14 (32.6%) | 21 (30.9%) |
| Flow-limiting angiographic complications | 26 (60.5%) | 39 (57.4%) |
| Major side branch occlusion | 25 (58.1%) | 35 (51.5%) |
| Distal embolization | 6 (14.0%) | 10 (14.7%) |
| Disruption of collateral flow | 0 (0.0%) | 1 (1.5%) |
| Slow flow/no-reflow | 0 (0.0%) | 1 (1.5%) |
| New regional wall motion abnormality or loss of viable myocardium | 8 (18.6%) | 11 (16.2%) |
| New regional wall motion abnormality | 7 (2.3%) | 10 (14.7%) |
| New loss of viable myocardium | 2 (4.7%) | 2 (2.9%) |

Note: More than one supporting imaging evidence of myocardial ischemia was present in some patients.


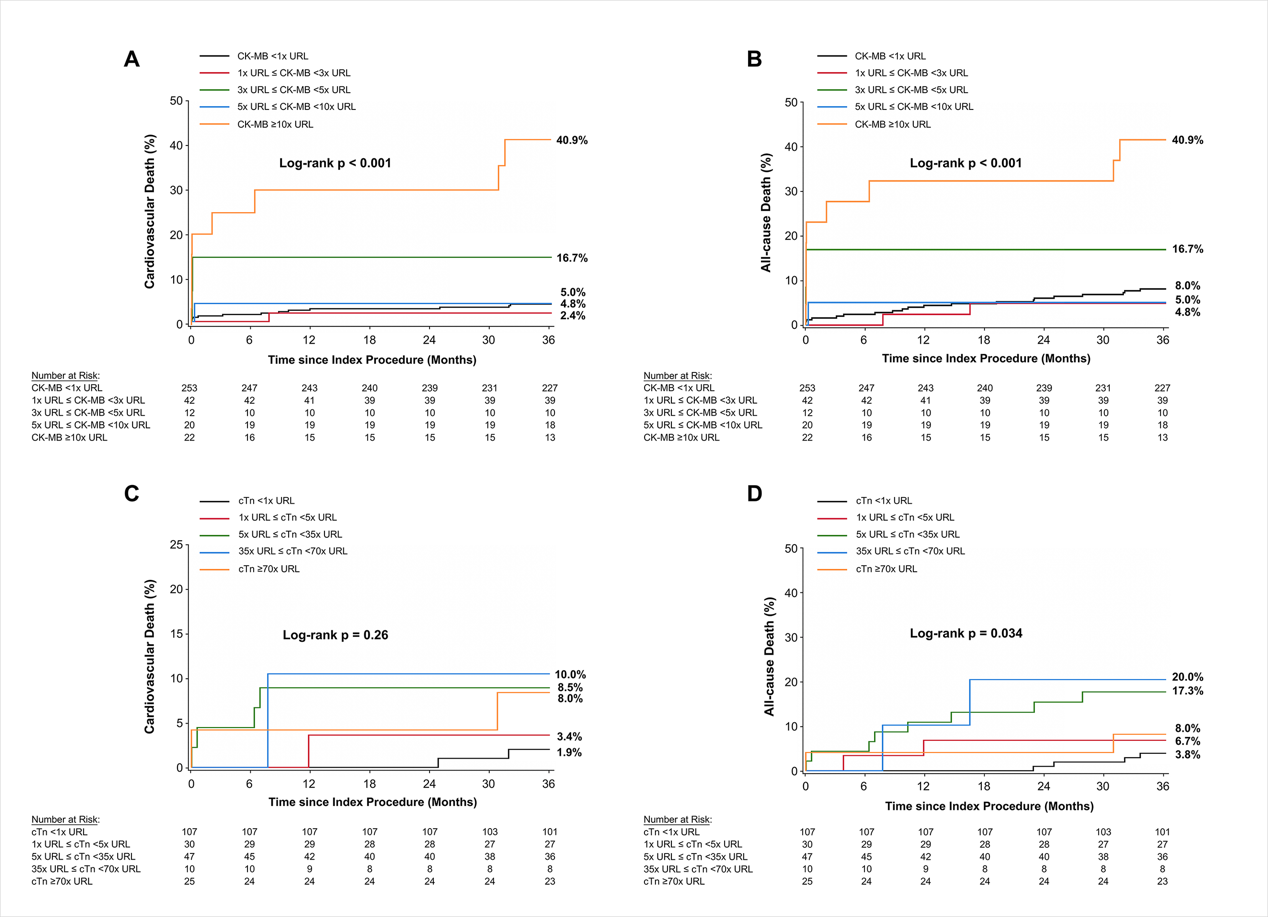


**Supplementary Figure 1. Time-to-event Curves for 3-year Cardiovascular and All-cause Death According to Absolute Rise in Post-PCI CK-MB and cTn Levels from Baseline.**

Kaplan-Meier curves for cardiovascular death (A and C) and all-cause death (B and D) according to different cutoffs of the absolute post-PCI incremental increases in CK-MB and cTnI levels from baseline, respectively.

URL = upper reference limit.
